# Ribosomal DNA as DAMPs Signal for MCF7 Cancer Cells

**DOI:** 10.3389/fonc.2019.00445

**Published:** 2019-05-30

**Authors:** Elena M. Malinovskaya, Elizaveta S. Ershova, Natalya A. Okorokova, Vladimir P. Veiko, Marina S. Konkova, Ekaterina A. Kozhina, Ekaterina A. Savinova, Lev N. Porokhovnik, Serguey I. Kutsev, Nataly N. Veiko, Svetlana V. Kostyuk

**Affiliations:** ^1^Research Centre for Medical Genetics (RCMG), Moscow, Russia; ^2^Biotechnology Research Center, Bach Institute of Biochemistry, Russian Academy of Sciences, Moscow, Russia

**Keywords:** DAMPs, human ribosomal gene, rDNA, TLR9, AIM2, cfDNA, oxidized cfDNA

## Abstract

**Introduction:** The cell free ribosomal DNA (cf-rDNA) is accrued in the total pool of cell free DNA (cfDNA) in some non-cancer diseases and demonstrates DAMPs characteristics. The major research questions: (1) How does cell free rDNA content change in breast cancer; (2) What type of response in the MCF7 breast cancer cells is caused by cf-rDNA; and (3) What type of DNA sensors (TLR9 or AIM2) is stimulated in MCF7 in response to the action of cf-rDNA?

**Materials and Methods:** CfDNA and gDNA were isolated from the blood plasma and the cells derived from 38 breast cancer patients and 20 healthy female controls. The rDNA content in DNA was determined using non-radioactive quantitative hybridization. In order to explore the rDNA influence on MCF7 breast cancer cells, the model constructs (GC-DNAs) were applied: pBR322-rDNA plasmid (rDNA inset 5836 bp long) and pBR322 vector. ROS generation, DNA damage, cell cycle, expression of TLR9, AIM2, NF-kB, STAT3, and RNA for 44 genes affecting the cancer cell viability were evaluated. The methods used: RT-qPCR, fluorescent microscopy, immunoassay, flow cytometry, and siRNA technology.

**Results:** The ratio R = cf-rDNA/g-rDNA for the cases was higher than for the controls (median 3.4 vs. 0.8, *p* < 10^−8^). In MCF7, GC-DNAs induce a ROS burst, DNA damage response, and augmentation of NF-kB and STAT3 activity. The number of the apoptotic cells decreases, while the number of cells with an instable genome (G2/M– arrest, micronuclei) increase. Expression of anti-apoptotic genes (*BCL2, BCL2A1, BCL2L1, BIRC3, MDM2*) is elevated, while expression of pro-apoptotic genes (*BAX, BID, BAD, PMAIP1, BBC3*) is lowered. The cells response for pBR322-rDNA is much more intense and develops much faster, than response for pBR322, and is realized through activation of TLR9- MyD88 - NF-kB- signaling. This difference in response speed is owing to the heightened oxidability of pBR322-rDNA and better ability to penetrate the cell. Induction of TLR9 expression in MCF7 is followed by blocking AIM2 expression.

**Conclusion:** (1) Ribosomal DNA accumulates in cfDNA of breast cancer patients; (2) Cell free rDNA induce DNA damage response and stimulates cells survival, including cells with an instable genome; (3) Cell free rDNA triggers TLR9- MyD88- NF-kB- signaling, with significantly repressing the expression of AIM2.

## Introduction

The operation of the innate immune system is based on sensors of different nature, termed pattern recognition receptors (PRRs). The PRRs recognize foreign molecules of viruses and bacteria. These sensors trigger multiple signaling cascades, converging on the production of type I interferons and proinflammatory cytokines (1). The PRRs also interact with biomolecules, which originate from the endogenous damaged and dying cells. The products of damaged cells, which are recognized by PRRs, are termed alarmins, or damage-associated molecular pattern (DAMPs) signals. The DAMP includes a very wide variety of biomolecules and low-molecular compounds (2, 3). Nucleic acids, which are released by damaged and dying cells, are also recognized by some PRRs and belong to the DAMPs family (4, 5). These DAMPs enter into the composition of cell-free DNA (cfDNA) and underlie the biological activity of cfDNA.

DAMPs in cancer have attracted growing interest in recent years. DAMPs are known to stimulate the immune cells. This results in exacerbation of the antitumor response (6, 7). However, DAMPs affect the physiology of the cancer cell itself (5, 8–14). Tumor cfDNAs could contribute to the progression of cancer and promote resistance to anticancer treatments (5, 15–18).

CfDNA includes various fragments of the genome of the dead cells. Obviously, not all cfDNA fragment exhibit the characteristics of DAMPs. In the literature, only one DAMPs for cancer cells is considered—mitochondrial DAMPs (mtDNA). The mtDNA is enriched with GC-pairs and includes unmethylated CpG motifs, which are TLR9 receptor agonists. The mtDNA was shown to activate TLR9 signaling during hypoxia to induce tumor growth (19–24).

**Figure 1:**
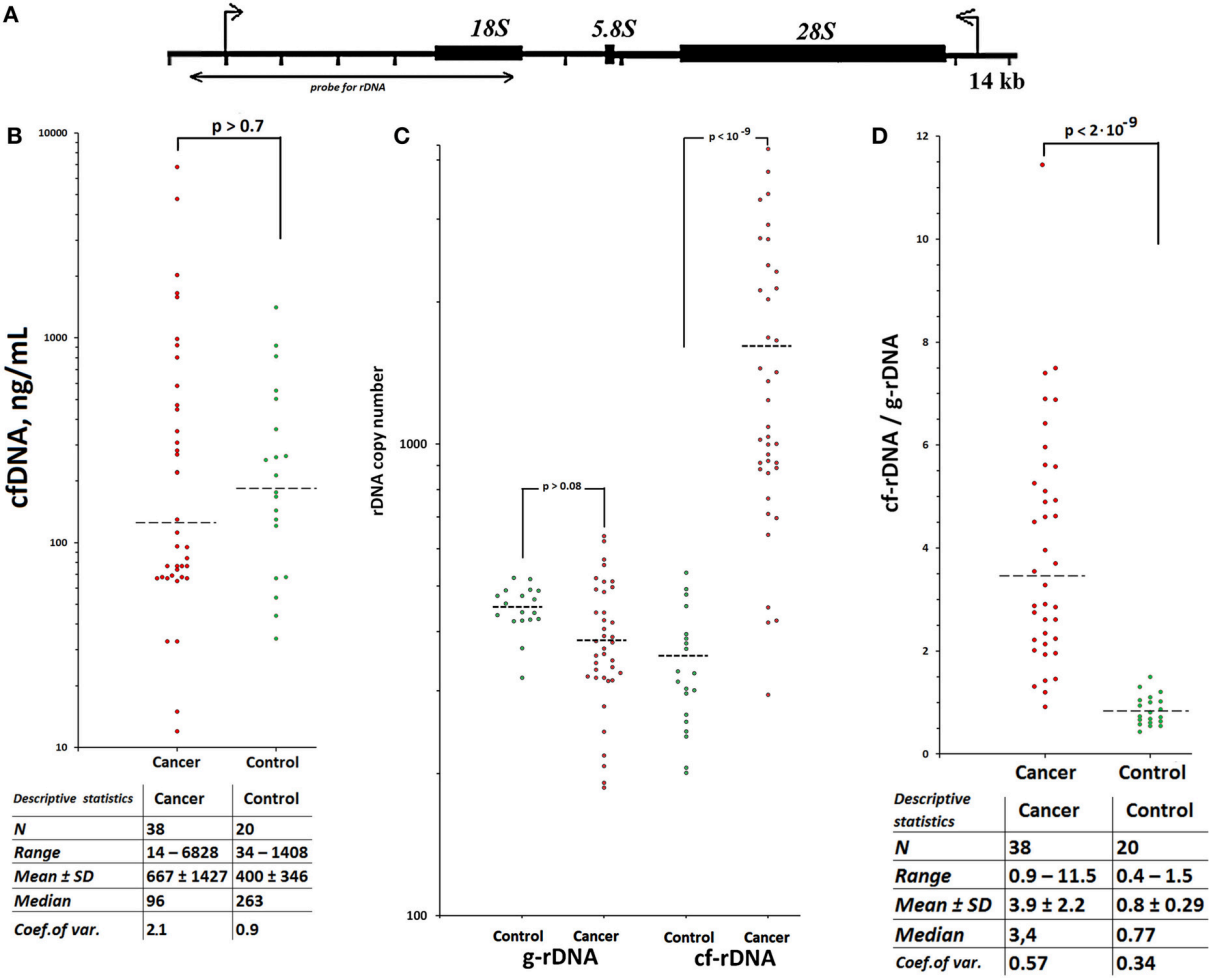
TR-rDNA accumulates in circulating cfDNA of patients with treated breast cancer. Scheme of the human ribosomal repeat. **(A)** Segment of the rDNA analyzed with NQH is shown. **(B)** CfDNA concentration for the two groups. Horizontal dotted lines represent the medians. Table below contains statistical data. Each point on the graph is an average of three independent experiments. The main contribution to the overall error of the experiment is made by the step of isolating DNA from the leukocytes. The total standard error was 10 ± 4%. **(C)** Ribosomal DNA copy number in the cell (genomic) DNA (g-rDNA) and in the plasma cell free DNA (cf-rDNA). Each point on the graph is an average of three independent experiments. The rDNA content was calculated using NQH (44). The total standard error was 11 ± 8%. Two groups do not differ in the content of rDNA in gDNA, but significantly differ in the content of rDNA in cfDNA (the data for the U-test are given). **(D)** The ratio R = cf-rDNA/g-rDNA; cf-rDNA and g-rDNA are the content of rDNA in cfDNA and gDNA of the same person. Table below contains statistical data. Two groups significantly differ in the ratio R (*U*-test).

Besides, the human genome contains some hundreds of copies of another GC-rich sequence—ribosomal DNA (25). The transcribed region of human ribosomal repeat (TR-rDNA) is a GC-rich sequence about 13.5 kb long, Figure 1A. Different areas of human TR-rDNA contain from 60% to 85% GC-pairs. TR-rDNA is accumulated in cfDNA, when a chronic process followed by an exaggerated cell death rate occurs (26). The TR-rDNA content is augmented several times within cfDNA of patients with rheumatoid arthritis (27), ischemic heart disease (26), employees occupationally exposed to heightened ionizing radiation background (28). One can assume that TR-rDNA is also accumulated in cfDNA of cancer patients during the therapies, which result in an elevated cell death rate.

The potential biological activity of TR-rDNA is highlighted by the presence of specific antibodies to rDNA in blood serum of healthy controls (29). TR-rDNA fragments demonstrated promoting activity with regard to human mesenchymal stem cells, human umbilical vein cells (30) and rat cardiomyocytes (31). TR-rDNA can be also expected to materially affect the physiology of cancer cells. Cancer cells express well-known PRRs—DNA sensors, such as absent in melanoma 2 (AIM2) (32–37) and toll-like receptor 9 (TLR9) (38–41).

The DNA-sensors TLR9 and AIM2 recognizes cfDNA fragments, which penetrate the cells. However, these two sensors perform diametrically opposed functions in the cancer cell. TLR9 promotes survivability of cancer cells by activating the TLR9—MyD88 –NF-kB signaling pathway (39, 40), whereas AIM2 induces apoptosis in cancer cells being a part of the inflammasome. In MCF7 breast cancer cell lines, AIM2 induction promotes apoptosis through the mitochondrial pathway and expression of pro-apoptotic proteins (42). MCF7 cells also express TLR9, moreover, it occurs both on the cell surface and in the endosome (43). It is so far unclear, how these two opposite signaling pathways, which are activated by the two DNA-sensors that are able to recognize the same free DNA fragments in the cytoplasm, interact in the cancer cell.

Therefore, the major questions of this study were: (1) How does cell free rDNA content change in cancer; (2) What type of response in the MCF7 breast cancer cells is caused by TR-rDNA; and (3) What type of DNA sensors (TLR9 or AIM2) is stimulated in MCF7 in response to the action of TR-rDNA?

## Materials and Methods

### Subjects

Thirty-eight female breast cancer patients had applied for genetic tests in RCMG. Previously, patients were treated with chemotherapy or radiation therapy in oncology departments of Moscow clinics. The average age of the examined individuals was 44.2 ± 12.3 years. The control group consisted of 20 healthy females with no history of any cancer, age-matched to the patient group. There were no statistical differences between the examined groups in terms of smokers frequencies. The investigation was carried out in accordance with the latest version of the Declaration of Helsinki. The Regional Ethics Committee of RCMG approved it (approval #5). All participants signed an informed written consent to participate after the nature of the procedures had been explained to them.

### Cell Culture

ER/PR-positive MCF7 breast cancer cells were purchased at ATCC, Manassas, USA (Cat: HTB22). MCF7 cells were cultured in DMEM medium supplemented with 10% (v/v) fetal calf serum, 2 mM L-glutamine, 100 units/mL penicillin, and 100 μg/mL of streptomycin. Cells were grown in a humidified atmosphere with 5% CO2 in air at 37°C.

GC-DNAs were added to the cell culture medium. Unless otherwise noted, the GC-DNAs concentration in the medium was 50 ng/mL.

### DNA Isolation From the Blood

Five ml of the blood was collected from the peripheral vein with a syringe flushed with heparin (0.1 mL/5 mL blood) under strict aseptic conditions. Cells were removed from the blood by centrifugation at 460 × g. DNA was isolated from leukocytes (gDNA) and plasma (cfDNA) by the standard method (28). The DNA quantification is performed fluorimetrically using the PicoGreen dsDNA quantification reagent by Molecular Probes (Invitrogen, CA, USA). We use EnSpire equipment (Finland) at excitation and emission wavelengths of 488 and 528 nm, respectively. Relative standard error of the cfDNA concentration in the plasma was 10 ± 4%.

### Non-radioactive Quantitative Hybridization (NQH)

The method of quantitative non-radioactive hybridization was specified in details previously (28, 44). Briefly, the denatured gDNA or cfDNA samples (50 ng/mL) were applied to a prepared filter (Optitran BA-S85, GE healthcare). From 4 to 6 dots were applied per each sample. Six standard samples of the genomic DNA (50 ng/mL) with a known content of the rDNA were applied to the same filter, in order to plot a calibration curve for the dependence of the signal intensity on the number of rDNA copies in a particular sample. Lambda phage DNA (50 ng/mL) was also applied to the same filter in order to control the non-specific signal. The filter was heated at 80°C in vacuum for 1.5 h. For the detection of the human ribosomal repeat, the probe p(ETS-18S) (the fragment of rDNA 5.8-kb long, from −515 till 5321 relative to the transcription initiation point, HSU 13369, GeneBank) was used (Figure 1A). It was cloned into the vector pBR322. The rDNA-probe was biotinylated using the nick translation kit (Biotin NT Labeling Kit, Jena Bioscience GmbH). After hybridization was completed, the dried filter was scanned. In order to assay the hybridization outcome, special software was used (Images6, Research Center for Medical Genetics, Moscow). The software determined the dot location, measured the nearest background signal and calculated the integral dot intensity. Signals from several dots corresponding to the same sample were averaged. The rDNA content in a studied DNA sample was calculated using the calibration curve equation. Relative standard error was 11 ± 8%.

### Model GC-DNAs

**pBR322-rDNA:** plasmid DNA (10,197 bp) contains rDNA sequences (5836 bp, 73% GC) cloned into EcoRI site of pBR322 vector. The rDNA fragment cloned covers the positions from −515 to 5321 of human rDNA (Figure 1A).

**pBR322:** vector (4361 bp, 53% GC) served as a control for pBR322-rDNA. Plasmid pBR322 is the commercial product (Sigma-Aldrich).

**Figure 2:**
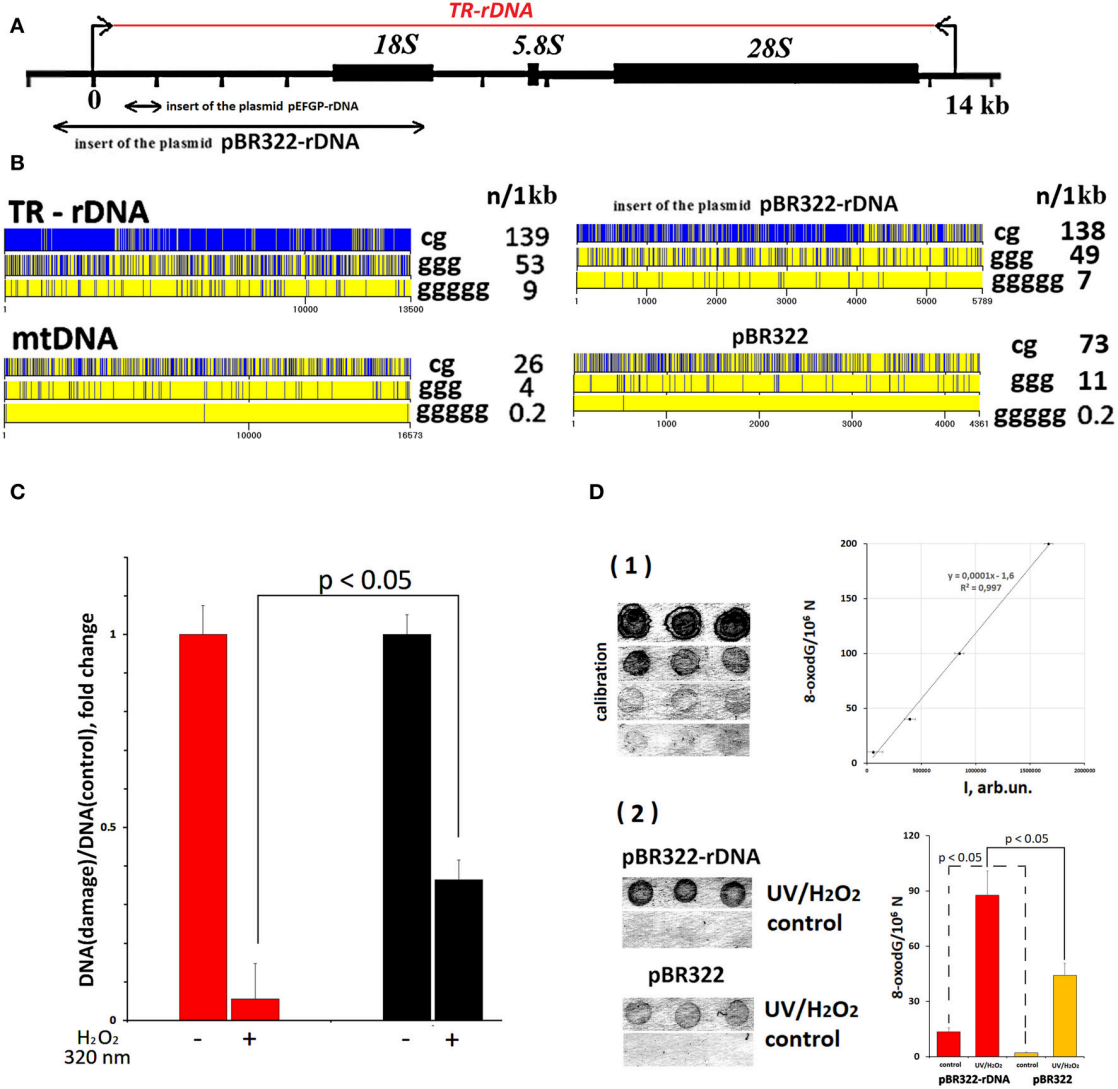
TR-rDNA sequence bears hallmarks of DAMPs. **(A)** Scheme of the TR-rDNA. Segments of the rDNA inserted into plasmids are shown. **(B)** Distribution of CpG motifs (TLR9 agonists) and dGn motifs (prone to the oxidation) within TR-rDNA, mtDNA, inserts of the plasmid pBR322-rDNA and vector pBR322. The digits indicate the nucleotide order number, while the vertical bar shows the motif location. The numbers on the right show the number of motifs in a region of DNA 1 1kb long. **(C)** Under the action of ROS (H_2_O_2_/320 nm), rDNA is oxidized largely than mtDNA. Parameter of rDNA and mtDNA damage was calculated by normalizing a damaged amplicon (hydrolyzed with Fpg) to intact amplicon. Each point on the graph is an average of three independent experiments. **(D)** Under the action of ROS (H_2_O_2_/320 nm), pBR322- rDNA is oxidized largely than pBR322. The method of immunoassay on nitrocellulose membrane using 8-oxodG antibodies conjugated with alkaline phosphatase was used. (1) Four standard samples of the oxidized genomic DNA (10 ng/dot) with a known content of 8-oxodG (was determined by ESI-MS/MS) were applied in order to plot a calibration curve for the dependence of the signal intensity on the content of 8-oxodG. (2) The samples of the oxidized and non-oxidized (control) pBR322 and pBR322-rDNA (10 ng/dot) were applied. The experiment was repeated twice.

**pEGFP-rDNA:** Plasmid DNA (5151 bp) contains GC-rich rDNA sequences (420 bp, 91.9% GC) cloned into BamH1 site of pEGFP-C1 vector. Cloned rDNA fragment covers positions from 601 to 1021b of human rDNA (Figure 2A).

**pEGFP:** plasmid pEGFP-C1 (pEGFP) (53.4 % GC) that contains the EGFP gene (http://www.bdbiosciences.com, GenBank accession number U55763) was used as control for pEGFP-rDNA.

All the plasmids are enriched with GC-pairs compared to human gDNA (42% GC). All the GC-DNA samples were subjected to the purification with EndoFree Plasmid Purification kit (www.qiagen.com). In order to prove that the observed response was caused exclusively by DNA, not by endotoxin residuals, additional experiments were set up. (1) A sample of plasmid DNA underwent complete hydrolysis down to nucleosides using DNA exonucleases and phosphatase. The resultant plasmid hydrolysates had no biological activity, which is intrinsic to intact DNA. (2) We analyzed the expression of TLR4 gene, which is always activated in the presence of the endotoxin. The samples of plasmid DNA induced no increase of TLR4 expression.

A question whether plasmid DNA requires linearization before adding to the culture medium was considered earlier (45). Our tests failed to show any difference between the rates of accumulation of intact GC-DNAs and their linearized forms in the cells. When the cells were present in the medium, the supercoiled circular plasmid DNA is rapidly hydrolyzed down to linear molecules of different length. On the basis of these data, we added non-linearized plasmids to the culture medium during the major experiments.

### Construction of Recombinant RNAi-ReadypSIREN-DNR-DsRed-Express Vectors

Two siRNA target sites for each genes were selected from coding sequence of AIM2 (AF024714.1) and TLR9 (NM_017442). To obtain short hairpin RNA oligonucleotides (Table 1) were synthesized and inserted into the BamHI and EcoRI sites of the RNAi-ReadypSIREN-DNR-DsRed-Express vector (BD Biosciences, Clontech, Mountain View, CA, www.clontech.com). For the control shRNA a plasmid pK encoding a luciferase siRNA was constructed (oligonucleotide provided by Clontech with pSIREN vector). Plasmids of pK-AIM2(1), pK-AIM2(2), pK-TL9(1), pK-TL9(2), and pK (negative control) were purified using EndoFree Plasmid Purification kit (www.qiagen.com) and confirmed by sequencing.

**Table 1:** Sequences of DNA for shRNA obtaining.

**Synthetic DNA sequence**	**Plasmid name**	**Position (mRNA)**
5′GATCCG**AACATCACTGATGAGGAAC**TTCAAGAGA**GTTCCTCATCAGTGATGTT**CTTTTTG GCTTGTAGTGACTACTCCTTGAAGTTCTCTCAAGGAGTAGTCACTACAAGAAAAACTTAA 5′	pK-AIM2(1)	290
5′GATCCg**GAATCTATCAGAGAAGGGT**TTCAAGAGA**ACCCTTCTCTGATAGATTC**cCTTTTTG GCCTTAGATAGTCTCTTCCCAAAGTTCTCTTGGGAAGAGACTATCTAAGGGAAAAACTTAA 5′	pK-AIM2(2)	670
5′GATCCG**TGACCATCGAGCCCAGCACC**TTCAAGAGA**GGTGCTGGGCTCGATGGTCA**CTTTTTG GCACTGGTAGCTCGGGTCGTGGAAGTTCTCTCCACGACCCGAGCTACCAGTGAAAAACTTAA 5′	pK-TLR9(1)	969
5′GATCCG**CCTCCGAGTGCTGGACCTGA**TTCAAGAGA**TCAGGTCCAGCACTCGGAGG**CTTTTTG GCGGAGGCTCACGACCTGGACTAAGTTCTCTAGTCCAGGTCGTGAGCCTCCGAAAAACTTAA	pK-TLR9(2)	1561
5′GATCC**GTGCGTTGCTAGTACC**AACTTCAAGAGATTTTTTACGCGTG GCACGCAACGATCATGGTTGAAGTTCTCTAAAAAATGCGCACTTAA5′	pK	

*Bold, sense and antisense strands*.

To confirm the efficiency of AIM2 and TL9 knockdown, RT-PCR analysis was conducted to detect mRNA level after transfection with plasmids pK-AIM2(1), pK-AIM2(2), pK-TL9(1), pK-TL9(2), and negative control (pK). The primer pairs used in this part of investigation:

AIM2(118 bp) F: GAAGCCCTTCACGTTTGAGACCCA; R: TGAATTTATCTTTCAGCAGTGTA

TLR9 (161 bp) F: AAATTGCCGCCGCTGCGACCAC; R: AGCCCACGGAACCAACTGGCATT.

### Analysis of rDNA and mtDNA Fragmentation

TR-rDNA analysis by qPCR method and primers for mtDNA and TR-rDNA are described earlier (44, 46). 8-oxoguanine DNA glycosylase (fpg) were used for gDNA hydrolysis under manufacturer's protocol (Merck). Parameter of DNA fragmentation was calculated by normalizing an fpg—hydrolyzed amplicon to intact (unhydrolyzed) amplicon (Figure 2C).

### 8-oxodG Levels in the Model GC-DNAs

The method for DNA oxidation was specified previously (47). Briefly, plasmids (100 ng/μL) were oxidized in 1% H_2_O_2_ solution with UV irradiation (λ = 320 nm) for 3 min at 25°C. Modified DNA was precipitated with 2 volumes of ethanol in the presence of 2 M ammonium acetate. The precipitate was washed twice with 75% ethanol, then dried and dissolved in water. Resulting DNA concentrations were assessed by the analysis of UV spectra.

The method for 8-oxodG quantitation was specified in details previously (48). Briefly, the DNA samples were applied to a prepared filter (Optitran BA-S85, GE healthcare). Three dots (10 ng/dot) were applied per each sample. Four standard samples of the oxidized genomic DNA (10 ng/dot) with a known content of 8-oxodG (was determined by ESI-MS/MS using AB SCIEX 3200 Qtrap machine) were applied to the same filter, in order to plot a calibration curve for the dependence of the signal intensity on the number of 8-oxodG copies in a particular sample. The filter was heated at 80°C in vacuum for 1.5 h. 8-oxodG antibody conjugated with alkaline phosphatase was used. Then the filter was placed into a solution of substrates for alkaline phosphatase NBT and BCIP. Upon the completion of reaction, the filter was washed with water and dried in the darkness. The dried filter was scanned. For the quantitative analysis of the dots, special software was used (Images6, RCMG, Moscow). Signals from several dots for the same sample are averaged. The 8-oxodG content in a studied sample is calculated using the calibration curve equation. Relative standard error was 15 ± 5%.

### Labeled Probe pBR322-rDNA^**red**^

Labeling of plasmid DNA was performed by nick translation using CGH Nick Translation Kit (Abbott Molecular) under manufacturer's protocol with slight modification. Solutions of plasmid DNA (3 μg/μL) were labeled with SpectrumRed. In the reaction mix, 50% of the dTTP was substituted with the labeled dUTP. About 20% of the fluorescent-labeled nucleotide was incorporated into the DNA, while unincorporated nucleotides were removed by ethanol precipitation. The fragment size was in 300–3,000 bp range as determined by electrophoresis in 1% agarose.

### Flow Cytometry (FCA)

Before FCA, cells were washed in Versene solution, than treated with 0.25 % trypsin under control of light microscopic observation. Cells were washed with culture media, then centrifuged and resuspended in PBS. Staining of the cells with various antibodies was performed as described below (47). Briefly, to fix the cells, the paraformaldehyde (Sigma) was added at a final concentration of 2% at 37°C for 10 min. Cells were washed with 0.5% BSA-PBS (x3) and permeabilized with 0.1% Triton X-100 (Sigma) in PBS for 15 min 4°C. Cells (~ 50 × 10^3^) were washed with 0.5% BSA-PBS and stained with 1 μg/mL FITC-γH2AX (Ser139) antibody (Temecula California), FITC-Ki-67 antibody. In other cases, cells were stained with PCNA, AIM2, TLR9, NF-κB (p65), and STAT3 antibodies (Abcam) for 3 h at 4°C, then again washed with 0.5% BSA-PBS and stained with 1 μg/mL secondary FITC- or PE-conjugated antibodies (Abcam) for 1 h at 4°C.

To quantify intracellular DNA, cells were treated with propidium iodide (PI) and RNAase A. To quantify the background fluorescence, we stained a portion of the cells with secondary FITC(PE)-conjugated antibodies only.Cells were analyzed at CyFlow Space (Partec, Germany).

### Quantification of mRNA

Total mRNA was isolated using RNeasy Mini kits (Qiagen, Germany), treated with DNAse I, and reverse transcribed by a Reverse Transcriptase kit (Sileks, Russia). The expression profiles were obtained using qRT-PCR with SYBR Green PCR Master Mix (Applied Biosystems). The mRNA levels were analyzed using the StepOnePlus (Applied Biosystems); the technical error was ~2%. See Table 2 below for the list of primers used (Sintol, Russia).

*B2M*2 (reference gene): F GCTGGGTAGCTCTAAACAATGTATTCA; R CATGTACTAACAAATGTCTAAAATGG. *TBP* (reference gene): F GCCCGAAACGCCGAATAT; R: CCGTGGTTCGTGGCTCTCT

**Table 2:** Primers list.

**Gene**	**Forward primer(5^**′**^-3^**′**^)**	**Reverse primer(5^**′**^-3^**′**^)**
*AKT1*	GTCATCGAACGCACCTTCCAT	AGCTTCAGGTACTCAAACTCGT
*AKT2*	AGGCACGGGCTAAAGTGAC	CTGTGTGAGCGACTTCATCCT
*AKT3*	AATGGACAGAAGCTATCCAGGC	TGATGGGTTGTAGAGGCATCC
*AMBRA*	GCCCAGACAGGACTCTCTTAG	TGAACACACTTGCCAGTCTTC
*BAD*	CCCAGAGTTTGAGCCGAGTG	CCCATCCCTTCGTCGTCCT
*BAX*	GGAGCTGCAGAGGATGATTG	GGCCTTGAGCACCAGTTTG
*BBC3*	GCCAGATTTGTGAGACAAGAGG	CAGGCACCTAATTGGGCTC
*BCL2*	GCCTTCTTTGAGTTCGGTGG	ATCTCCCGGTTGACGCTCT
*BCL2A1*	TACAGGCTGGCTCAGGACTAT	CGCAACATTTTGTAGCACTCTG
*BCL2L1*	CGACGAGTTTGAACTGCGGTA	GGGATGTCAGGTCACTGAATG
*BID*	ATGGACCGTAGCATCCCTCC	GTAGGTGCGTAGGTTCTGGT
*BIRC2*	GAATCTGGTTTCAGCTAGTCTGG	GGTGGGAGATAATGAATGTGCAA
*BIRC3*	AAGCTACCTCTCAGCCTACTTT	CCACTGTTTTCTGTACCCGGA
*BRCA1*	TGTGAGGCACCTGTGGTGA	CAGCTCCTGGCACTGGTAGAG
*BRCA2*	ACAAGCAACCCAAGTGTCAAT	TGAAGCTACCTCCAAAACTGTG
*CCND1*	GCTGCGAAGTGGAAACCATC	CCTCCTTCTGCACACATTTGAA
*CDKN1A*	GGAAGACCATGTGGACCTGT	ATGCCCAGCACTCTTAGGAA
*CDKN2A*	ATGGAGCCTTCGGCTGACT	TAACTATTCGGTGCGTTGGG
*HIF1A*	ATCCATGTGACCATGAGGAAATG	TCGGCTAGTTAGGGTACACTTC
*HUWE1*	TTGGACCGCTTCGATGGAATA	TGAAGTTCAACACAGCCAAGAG
*IFNG*	TCGGTAACTGACTTGAATGTCCA	TCGCTTCCCTGTTTTAGCTGC
*IL1B*	GGTGTTCTCCATGTCCTTTGTA	GCTGTAGAGTGGGCTTATCATC
*IL6*	AAATTCGGTACATCCTCGACGGCA	AGTGCCTCTTTGCTGCTTTCACAC
*IL8*	ACTGAGAGTGATTGAGAGTGGAC	AACCCTCTGCACCCAGTTTTC
*IL10*	AAGGCGCATGTGAACTCCC	ACGGCCTTGCTCTTGTTTTC
*MAP3K1*	CTACACGCAGTTGCAGTACAT	CAGCAGGATCTGGATCTCCC
*MAP4K4*	GAGCCACAGGTACAGTGGTC	AAGCCTTTTGGGTAGGGTCAG
*MDM2*	CAGTAGCAGTGAATCTACAGGGA	CTGATCCAACCAATCACCTGAAT
*MMP2*	CCCACTGCGGTTTTCTCGAAT	CAAAGGGGTATCCATCGCCAT
*MMP7*	GAGATGCTCACTTCGATGAGG	CCTAGACTGCTACCATCCGTC
*MYD88*	GGCTGCTCTCAACATGCGA	TGTCCGCACGTTCAAGAACA
*MTOR*	TCCGAGAGATGAGTCAAGAGG	CACCTTCCACTCCTATGAGGC
*NFKB1*	CAGATGGCCCATACCTTCAAAT	CGGAAACGAAATCCTCTCTGTT
*PMAIP1*	ACCAAGCCGGATTTGCGATT	ACTTGCACTTGTTCCTCGTGG
*RELA*	TAAGCAGAAGCATTAACTTCTCTGGA	CCTGCTTCTGTCTCTAGGAGAGTA
*RIG1*	GAGATTTTCCGCCTTGGCTAT	CCGTTTCACCTCTGCACTGTT
*STAT3*	GGGTGGAGAAGGACATCAGCGGTAA	GCCGACAATACTTTCCGAATGC
*STAT6*	GTTCCGCCACTTGCCAATG	TGGATCTCCCCTACTCGGTG
*STING*	CCAGAGCACACTCTCCGGTA	CGCATTTGGGAGGGAGTAGTA
*TIRAP*	ATGGTGGCTTTCGTCAAGTCA	TCAGATACTGTAGCTGAATCCCG
*TNFa*	CAGCCTCTTCTCCTTCCTGAT	GCCAGAGGGCTGATTAGAGA
*TP53*	TTTGGGTCTTTGAACCCTTG	CCACAACAAAACACCAGTGC
*VEGFA*	AGGCCAGCACATAGGAGAGA	TTTCTTGCGCTTTCGTTTTT

### Fluorescence Microscopy (FM)

Cell images were obtained using the AxioScope A1 microscope (Carl Zeiss).

#### Immunocytochemistry

MCF7 cells were fixed in 3% formaldehyde (4°C) for 20 min, washed with PBS and then permeabilized with 0.1% Triton X-100 in PBS for 15 min at room temperature, followed by blocking with 0.5% BSA in PBS for 1 h and incubated overnight at 4°C with the γH2AX, TLR9, AIM2, NF-kB(p65), STAT3 antibody (Abcam). After washing with 0.01% Triton X-100 in PBS MCF7 cells were incubated for 2 h at room temperature with the FITC/PE goat anti-mouse IgG, washed with PBS and then stained with DAPI.

#### Intracelullar Localization of Labeled pBR322-rDNA Fragments and DCF

Labeling of pBR322-rDNA was performed by nick translation using CGH Nick Translation Kit (Abbott Molecular) under manufacturer's protocol. MCF7 cell cultures were pretreated with 5μM of H2DCFH-DA (Molecular Probes/ Invitrogen, CA, USA) for 5 min, then labeled pBR322-rDNA ^***red***^ were added to the cultivation media for further 30 min. The cells were washed three times with PBS and immediately photographed.

#### Analysis of Genomic Instability

Before treatment with DNA probes, cells were grown for 24 h in slide flasks. The GC-DNAs fractions were added to cultivation media for 48 h. Cells were fixed in 3% formaldehyde (4°C) for 20 min washed with PBS and stained with 2 μg/mL DAPI. Approximately 1,000 cells were investigated for the presence of micronuclei, nuclear buds and nuclear bridges as described (49).

#### Nuclear Fragmentation

Was examined by Hoechst 33342 (Sigma) staining (10 μg/mL) for 10 min at 37°C. 1,000 cells were investigated for the presence of the damaged nuclei.

#### Comet Assay

The degree of the DNA damage was analyzed by means of the DNA-comet assay with CometAssay™ Reagent Kit for Single Cell Gel Electrophoresis Assay (Trevigen, Inc. 8405 Helgerman Ct. Gaithersburg, MD 20877). Average comet tails were selected for the calculation. One hundred nuclei per each slide were analyzed. The software package CometScore v. 1.5 (supplied by “TriTek Corp.” http://tritekcorp.com) was used for the comet assay performance.

### ROS Assay

The cells were analyzed using total fluorescence assay in the 96-well plate format at λex = 488 nm and λem = 528 nm (EnSpire equipment, Finland). The cultivation medium was replaced by 5 μm H2DCFH-DA (Molecular Probes/Invitrogen, CA, USA) in PBS solution and a relative fluorescence intensity increase was detected at 37°C. 8 (4 × 2) repeated measurements were provided for each GC-DNA and 16 for the control. The mean absolute intensities were divided by the average value of the intensity corresponding to t = 0, obtaining the values of I_0_. The graphs are presented in the coordinates I–time.

### Statistics

All reported results for qPCR, PT-qPCR, immunoassay and FCA were reproduced at least three times as independent biological replicates. The significance of the observed differences was analyzed using non-parametric Mann- Whitney U-tests. The data were analyzed with StatPlus2007 Professional software (http://www.analystsoft.com/). All *p*-values considered statistically significant at p < 0.05.

## Results

### TR-rDNA Is Accumulated in Circulating cfDNA of Breast Cancer Patients

Circulating cfDNA and gDNA were isolated from blood plasma and leukocytes of 38 female breast cancer patients, who received radiation therapy or chemotherapy, and 20 healthy female controls. The levels of cfDNA in plasma of cases and healthy controls were virtually equal (Figure 1B, see the table for descriptive statistics). The TR-rDNA content in cfDNA and gDNA was determined using non-radioactive quantitative hybridization (NQH) of the samples with biotinylated DNA-probe pBR322-rDNA (Figure 1A). Two groups do not differ in the content of rDNA in the gDNA, but significantly differ in the content of rDNA in cfDNA (Figure 1C). The ratio of TR-rDNA content in cfDNA to that in gDNA (index R = cf-rDNA/g-rDNA) in the patient group was significantly higher than in the healthy controls. In 33 of 38 patients (87%), the index R was higher than in the control group (Figure 1D, see the table for descriptive statistics).

### TR-rDNA Sequence Bears Hallmarks of DAMPs

Analysis of TR-rDNA sequence revealed two peculiarities, which enable considering TR-rDNA as a potential DAMPs molecule (Figure 2B).

First, TR-rDNA carries many unmethylated CpG motifs. The DNA regions with these motifs are ligands for TLR9 DNA-sensors (50). The density of CpG motifs along TR-rDNA is several times higher, than in mitochondrial DNA (mtDNA). MtDNA is known to be a generally recognized ligand for TLR9 (21).

Second, TR-rDNA contains many (dG)_n_ motifs (Figure 2B). The nucleoside dG inside (dG)_n_ has the lowest oxidation potential among all nucleosides in DNA (51). One may suppose that TR-rDNA is prone to oxidation. The oxidation of cfDNA drastically increases its penetration into the cancer cells. In the cytoplasm, cfDNA can potentially stimulate intracellular DNA sensors, for example, AIM2, STING, RIG and intracellular TLR9. It was also found that oxidized DNA stimulates DNA damage response in the cells (47).

To confirm the increased susceptibility of TR-rDNA to oxidation, we compared the efficiency of oxidation of TR-rDNA and mtDNA in gDNA isolated from blood leukocytes of healthy young donor (Figure 2C). In order to quantify the oxidation marker 8-oxodG, qPCR technique and enzyme 8-oxo-guanine DNA glycosylase (Fpg) were used. Because of DNA hydrolysis with Fpg, 8-oxodG is eliminated from the DNA strand, and this DNA is no longer a template for PCR (excluded from the analysis). We examined the regions of mtDNA and TR-rDNA having approximately the same dG count. After gDNA exposure to ROS in solution [1% hydrogen peroxide/ irradiation at 320 nm (47)], the number of fragments not containing 8-oxodG was several times lower in the case of rDNA fragment (Figure 2C). Therefore, TR-rDNA was oxidized in a much greater degree, than mtDNA.

Thus, one would expect that (1) TR-rDNA stimulates the well-studied signaling pathway TLR9–MyD88–NF-kB, and (2) TR-rDNA oxidizes easily on the surface of the cancer cells and after oxidation, it can induce the DNA damage response (DDR) in the cell.

### Model GC-DNA Fragments

CfDNA contains various GC-DNAs and is substantially damaged (48). Therefore, cfDNA is not a suitable object to explore the TR-rDNA action on the cancer cells. In order to find out, if TR-rDNA fragments can affect the physiology of cancer cells, we investigated a model system: MCF7 cancer cell line culture exposed to plasmids pBR322-rDNA and pBR322. pBR322 - rDNA (10197 bp) contains rDNA sequences (5836 bp, 73% GC) cloned into pBR322 vector (4361 bp, 53% GC). The cloned rDNA fragment covers positions from −515 to 5,321 of human TR-rDNA (Figure 2A). pBR322 served as a control. Vector pBR322 is similar to mtDNA in terms of CpG and (dG)_n_ motif content and drastically differs from TR-rDNA (Figure 2B). Lengthy easily oxidizable (dG)_n_ motifs can be found in pBR322-rDNA much more frequently, than in pBR322 or mtDNA. Thus, pBR322-rDNA and pBR322 plasmids are models for different GC-DNAs of human genome, which could affect cancer cells in different ways.

The elevated oxidability of pBR322-rDNA plasmid was proved using immunoassay (Figure 2D). The technique enables quantification of the oxidation marker 8-oxodG in DNA. Despite the identical conditions of plasmid isolation and storage, the intact pBR322-rDNA was more strongly oxidized, than the vector (fourteen 8-oxodG/10^6^ N vs. two 8-oxodG/10^6^ N). After oxidation in solution [1% hydrogen peroxide/radiation at 320 nm (47)] 8-oxodG was found approximately in 22% of pBR322 molecules and in 51% of pBR322-rDNA molecules. Hence, pBR322-rDNA, like TR-rDNA, contains an easily oxidizable motifs, which have very low oxidation potentials.

### TR-rDNA Stimulates TLR9—NF-kB Signaling and Blocks AIM2-Signaling

#### The Influence of GC-DNAs on TLR9 and AIM2 Expression

The MCF7 cells express TLR9 and AIM2 (Supplementary Figure 1). The genes *AIM2* and *TLR9* respond to a change in the time of cultivation in different ways: *TLR9* expression considerably increases with the time, while *AIM2* expression drastically decreases. One of the causes of the increase in TLR9 expression can be an increase in cfDNA content with time of the cell cultivation (Supplementary Figure 2).

In order to understand, if the expressions of genes *AIM2* and *TLR9* are related, we used a technique of inhibiting gene expression with siRNA (Supplementary Figure 3). We found that inhibition of *TLR9* expression with the siRNAs considerably elevates *AIM2* expression, especially at the level of RNA amount. Inhibition of *AIM2* expression affects *TLR9* expression to a much smaller degree.

**Figure 3:**
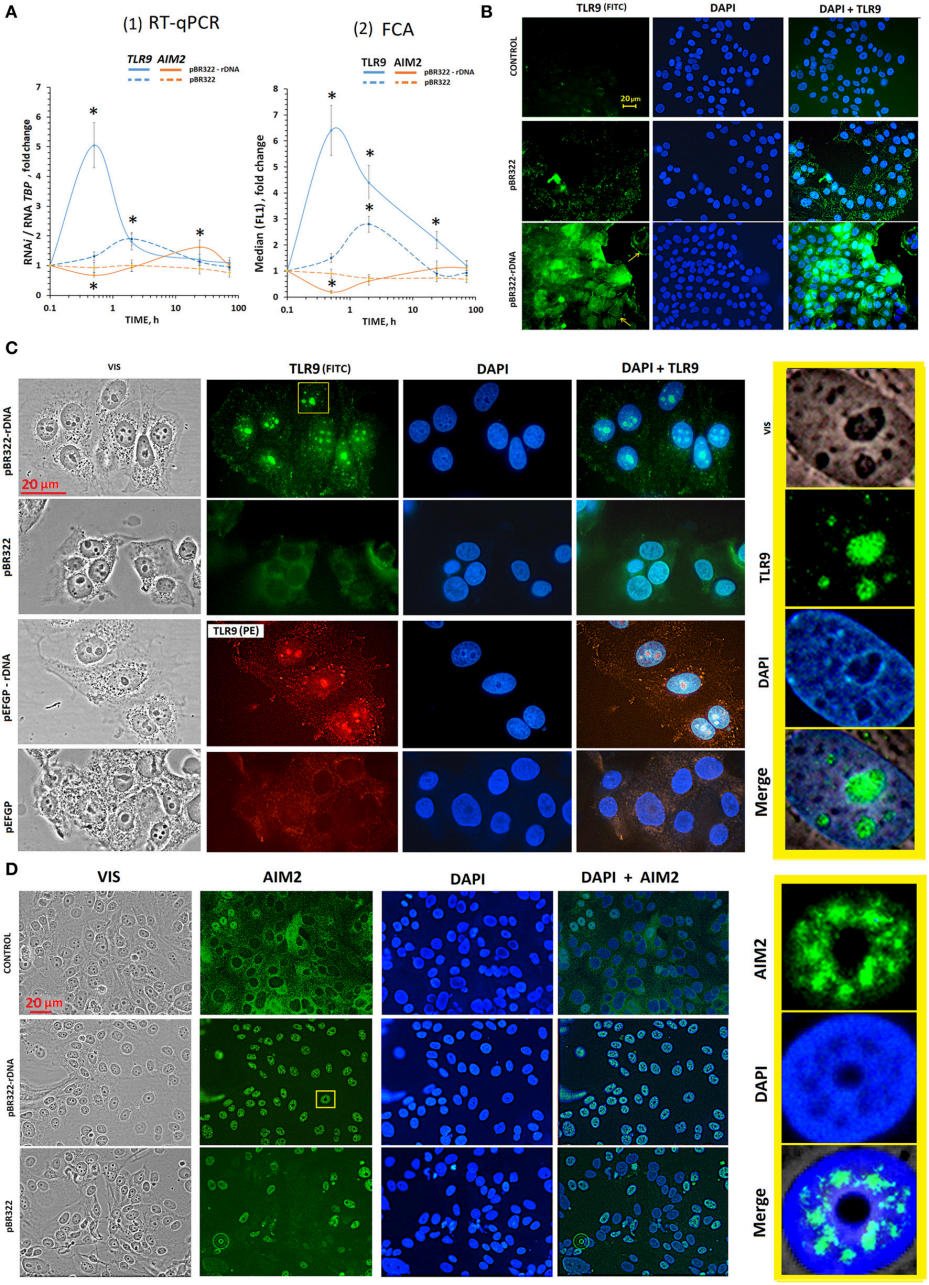
The influence of GC-DNAs on *TLR9* and *AIM2* expression. **(A)** (1) RT-qPCR. The dependence of the RNA *TLR9* or RNA *AIM2* on the duration of the cultivation of the cells with pBR322–rDNA and pBR322. (2) FCA. The dependence of proteins TLR9 or AIM2 content on the duration of the cultivation of the cells with pBR322–rDNA and pBR322. Description of the definition of proteins by FCA is given in Supplementary Material. Each point on the graph is an average of four independent experiments. *p < 0.05 (differences with control are significant). **(B)** FM of the cells stained to TLR9. The cells were cultured for 24 h, then plasmid pBR322 or pBR322-rDNA was added and the cells were cultured for 1 h. **(C)** FM of the cells stained with TLR9 (FITC) and with TLR9(PE) antibodies. The cells were cultured for 24 h, then the cells were treated with pBR322–rDNA and pBR322 (green) or pEGFP and pEGFP-rDNA (red) for 24 h. The right photo—is an enlarged image of the selected area. **(D)** FM of the cells stained with AIM2 (FITC) antibodies. The cells were cultured for 24 h, then plasmids pBR322 and pBR322-rDNA were added and the cells were cultured for 1 h. All the FM-experiments **(B,C)** were repeated at least three times.

Plasmid pBR322—rDNA (50 ng/mL) induced a several times increase of RNA *TLR9* and TLR9 protein already in 30 min [Figure 3A (1 and 2), blue lines]. In 2 h, the content of RNA *TLR9* and TLR9 protein decreased, but still remained higher than the baseline within the following 48 h. pBR322 also induced expression of *TLR9*, but to a smaller degree than pBR322—rDNA (*p* < 0.05 for TLR9 protein at 0.5, 2, and 24 h [Figure 3A(2); *p* < 0.05 for RNA *TLR9* at 0.5 h Figure 3A(1)].

When inducing *TLR9* expression with pBR322—rDNA plasmid, we observed a significant inhibition of AIM2 expression at the levels of both RNA and protein (Figure 3A, brown lines). When pBR322 was used, the contents of RNA *AIM2* and AIM2 protein also decreased, but the changes were less pronounced (*p* < 0.05 for AIM2 protein at 0.5 h [Figure 3A(2); *p* < 0.05 for RNA *AIM2* at 0.5 h Figure 3A(1)].

Plasmid pBR322—rDNA also affects the amount of RNA of another DNA-sensor: stimulator of interferon genes (STING*)*. In 2 h, the level of RNA *STING* elevated six-fold, but in 48 h decreased below the control baseline (line 44, Table 3). In case of the vector pBR322, the amount of RNA *STING* increased by 80% as late as in 48 h (the effect is less than in the case of pBR322—rDNA; *p* < 0.05). Both plasmids had virtually no effect on the level of RNA of DNA-sensor retinoic acid-inducible gene 1 (RIG1) (line 43, Table 3).

**Table 3:** The changes in expression levels of selected mRNAs after exposure of MCF7 cells to either pBR322 or pBR322-rDNA.

		**pBR322-rDNA**		**pBR322**	
		**2 h**	**48 h**	**2 h**	**48 h**
**DOUBLE STRAND BREAK DNA REPAIR**					
1	*BRCA1*	2.0 ± 0.1^*^	3.1 ± 0.1^*^	1.9 ± 0.6^*^	4.2 ± 0.5^*^
2	*BRCA2*	1.3 ± 0.1	1.0 ± 0.1	0.7 ± 0.1	0.9 ± 0.1
**ANTI-APOPTOTIC**					
3	*BCL2*	2.5 ± 0.2^*^	4.9 ± 0.3^*^	1.1 ± 0.3	3.5 ± 0.5^*^
4	*BCL2A1 (Bfl-1/A1)*	2.8 ± 0.2^*^	2.5 ± 0.3^*^	1.2 ± 0.3	2.3 ± 0.3^*^
5	*BCL2L1 (BCL-X)*	1.5 ± 0.2^*^	2.1 ± 0.3^*^	1.0 ± 0.3	1.5 ± 0.1^*^
6	*BIRC3 (c-IAP1)*	2.0 ± 0.1^*^	0.8 ± 0.1	1.2 ± 0.2	4.0 ± 0.4^*^
7	*BIRC2 (c-IAP2)*	1.4 ± 0.1	1.8 ± 0.1^*^	1.2 ± 0.2	2.5 ± 0.1^*^
8	*MDM2*	2.7 ± 0.1^*^	1.8 ± 0.2^*^	1.8 ± 0.2^*^	2.8 ± 0.3^*^
**TUMOR SUPPRESSORS**					
9	*HUWE1*	0.1 ± 0.2^°^	0.8 ± 0.1	0.6 ± 0.3	1.0 ± 0.1
10	*AMBRA*	0.4 ± 0.1^°^	1.4 ± 0.1	0.8 ± 0.3	0.9 ± 0.2
**REGULATOR OF OXYGEN HOMEOSTASIS**					
11	*HIF1A*	4.3 ± 0.2^*^	1.5 ± 0.1	0.9 ± 0.3	0.9 ± 0.2
**NFκB PATHWAY**					
12	*MAP4K4*	2.2 ± 0.2^*^	1.3 ± 0.2	1.1 ± 0.1	1.5 ± 0.3
13	*MYD88*	2.5 ± 0.2^*^	2.0 ± 0.2^*^	1.2 ± 0.1	1.3 ± 0.2
14	*NFKB1*	2.6 ± 0.2^*^	1.9 ± 0.2^*^	0.8 ± 0.2	0.8 ± 0.4
15	*TIRAP*	2.0 ± 0.2^*^	2.2 ± 0.2^*^	0.8 ± 0.2	2.1 ± 0.3^*^
16	*RELA*	1.7 ± 0.2^*^	2.1 ± 0.2^*^	0.8 ± 0.3	2.2 ± 0.2^*^
17	*MAP3K1*	1.3 ± 0.2	1.4 ± 0.3	1.0 ± 0.3	1.3 ± 0.3
**CYTOKINES. CHEMOKINES AND RECEPTORS**					
18	*IL10*	3.8 ± 0.3^*^	1.5 ± 0.1^*^	3.2 ± 0.3^*^	5.3 ± 0.4^*^
19	*IFNG*	2.3 ± 0.3^*^	1.8 ± 0.2^*^	1.0 ± 0.1	1.9 ± 0.1^*^
20	*IL6*	1.4 ± 0.3	3.5 ± 0.3^*^	1.0 ± 0.3	0.8 ± 0.2
21	*IL8*	0.9 ± 0.2	3.4 ± 0.4^*^	0.7 ± 0.2	0.6 ± 0.4
22	*TNFa*	3.9 ± 0.4^*^	2.3 ± 0.3^*^	1.1 ± 0.2	0.5 ± 0.3
23	*IL1B*	0.4 ± 0.1^°^	1.6 ± 0.2	0.6 ± 0.3	1.4 ± 0.4
**STAT FAMILY**					
24	*STAT3*	2.2 ± 0.2^*^	1.6 ± 0.1^*^	1.1 ± 0.2	2.0 ± 0.2^*^
25	*STAT6*	1.3 ± 0.2	2.4 ± 0.3^*^	1.0 ± 0.2	3.1 ± 0.3^*^
26	*MMP2*	1.6 ± 0.2	1.1 ± 0.2	0.4 ± 0.1	0.3 ± 0.1
27	*MMP7*	1.7 ± 0.1^*^	1.8 ± 0.2^*^	1.0 ± 0.1	2.6 ± 0.2
**GROWTH FACTORS**					
28	*VEGFA*	2.3 ± 0.2^*^	1.8 ± 0.4^*^	1.0 ± 0.2	1.4 ± 0.1
**AKT/MTOR SIGNALING PATHWAYS**					
29	*MTOR*	9.4 ± 0.5^*^	3.5 ± 0.3^*^	0.5 ± 0.2	0.4 ± 0.2
30	*AKT1*	4.2 ± 0.3^*^	2.5 ± 0.2^*^	0.4 ± 0.2	0.5 ± 0.1
31	*AKT2*	3.6 ± 0.3^*^	1.5 ± 0.2^*^	0.8 ± 0.3	1.0 ± 0.3
32	*AKT3*	3.4 ± 0.2^*^	1.6 ± 0.1^*^	0.6 ± 0.2	0.8 ± 0.2
**CELL CYCLE CHECKPOINT AND CELL CYCLE ARREST**					
33	*CDKN2A*	3.0 ± 0.2^*^	1.1 ± 0.3	2.1 ± 0.1^*^	1.3 ± 0.3
34	*CDKN1A*	1.5 ± 0.1^*^	2.2 ± 0.2^*^	1.7 ± 0.2^*^	2.2 ± 0.2^*^
35	*TP53*	1.7 ± 0.2^*^	1.5 ± 0.2^*^	2.1 ± 0.3^*^	4.6 ± 0.2^*^
36	*CCND1 (CyclinD1)*	1.2 ± 0.3	1.6 ± 0.3	1.3 ± 0.2	1.2 ± 0.1
**PRO-APOPTOTIC**					
37	*BAX*	0.3 ± 0.2^°^	0.7 ± 0.1	1.3 ± 0.2	0.9 ± 0.3
38	*BID*	0.7 ± 0.2	3.1 ± 0.4^*^	0.4 ± 0.1^°^	0.2 ± 0.2^°^
39	*BAD*	0.4 ± 0.2^°^	2.4 ± 0.2^*^	0.3 ± 0.1^°^	0.2 ± 0.2^°^
40	*PMAIP1(NOXA)*	0.6 ± 0.1^°^	1.7 ± 0.2^*^	0.7 ± 0.1	0.3 ± 0.1^°^
42	*BBC3(PUMAdelta)*	0.7 ± 0.2	1.3 ± 0.2	0.2 ± 0.2^°^	0.3 ± 0.2^°^
**CYTOPLASMIC DNA RECEPTORS**					
43	*RIG1*	1.1 ± 0.2	1.3 ± 0.2	1.0 ± 0.3	1.3 ± 0.3
44	*STING*	6.2 ± 0.5	0.6 ± 0.2	0.8 ± 0.2	1.8 ± 0.3

Relative levels of expression are averages for three biological replicates and a standard deviation.

*,° *p < 0.05—against control cells, non-parametric U-test*.

#### The Influence of GC-DNAs on the Location of TLR9 and AIM2 in MCF7

The experiments were carried out on cells, which had been cultivated for 24 h. After 24 h of cultivation, the level of TLR9 in control cells was still quite low (Figure 3B and Supplementary Figure 1A). Weak signals were located in the cytoplasm. The level of AIM2 was, on the contrary, high yet (Supplementary Figure 1B). In most cells, the signals were located in the cytoplasm. In some cells, AIM2 was detected in the nuclei (Figure 3D).

**TLR9**. As early as in 30 min of the exposure to pBR322—rDNA, a significant increase in the protein content occurred as compared to the control cells (Figure 3B). The signals were located along the perimeter of the cells (yellow arrows in Figure 3B) and in the cytoplasm. This observation suggests expression of TLR9 both on the cell surface and inside the cells. In 24 h after adding pBR322—rDNA to the medium, the picture considerably changed, Figure 3C. Staining intensity along the perimeter of the cells and in the cytoplasm substantially decreased. The signals for TLR9 were found located in the nucleus, namely, in the nucleolar area, where ribosomal genes were located, Figure 3C. The vector pBR322 also induced TLR9 expression on the surface and inside the cells in 30 min of exposure, however the signals were not so numerous. 24 h later, signals disappeared on the cell surface. However, in contrast to pBR322—rDNA, the vector did not induce accumulation of TLR9 in the nucleolus.

This interesting fact of TLR9 accumulation in nucleoli was proved by experiments on another plasmid pair– vector pEFGP and plasmid pEFGP-rDNA. Cloned rDNA fragment covers positions from 601 to 1,021 b of human rDNA (Figure 2A). Antibodies labeled with PE were used as secondary antibodies, Figure 3C. Like in case of pBR322—rDNA, we observed accumulation of TLR9 in the nucleolus in 24 h of cell cultivation with pEFGP-rDNA. Vector pEFGP, like vector pBR322, did not induce accumulation of TLR9 in the nucleoli.

Thus, TR-rDNA fragments in plasmid pBR322—rDNA and pEFGP-rDNA considerably induced TLR9 expression in MCF7 cells resulted in accumulation of TLR9 protein in the cell nucleoli. Vectors did not exhibit this effect.

**AIM2**. In consequence of the exposure to plasmid pBR322—rDNA (1 or 24 h) location of AIM2 protein altered considerably (Figure 3D). As compared to the control cells, the protein was not detected in the cytoplasm, but migrated inside the nucleus. In the nucleus, AIM2 was located as compact structures in certain areas. The nucleoli are distinctly contrasted in the photo, and one can see that AIM2 never penetrated these compartments. In the presence of vector pBR322, AIM2 migrated to nuclei of approximately a half of the cells. The other cells expressed virtually no AIM2.

#### Activation of NF-kB Transcription Factor in the Presence of GC-DNAs

The experiments were carried out on the cells, which had been cultivated for 24 h. Using RT-qPCR, we explored changes at the level of RNA of some genes, principal players of this signaling pathway, under the action of plasmids pBR322—rDNA and pBR322 (Table 3, lines 12–17). As early as 30 min after the beginning of action, the contents of RNA for genes *NFKB1, MYD88, TIRAP*, and *RELA* increase and remain elevated in 48 h of cultivation. The vector pBR322 also induces transcription of these genes, but later and to a smaller degree. Activation of NF-kB factor in the presence of TR-rDNA was also proved by the data of an increase in the content of RNA for cytokines genes (*IFNG, TNF- alpha, IL6, IL8*), transcription of which is regulated by the factor NF-kB. The vector pBR322 induced a reliable increase in the amount of RNA *IFNG* only, and just after a long-lasting exposure (Table 3, lines 19–22).

**Figure 4:**
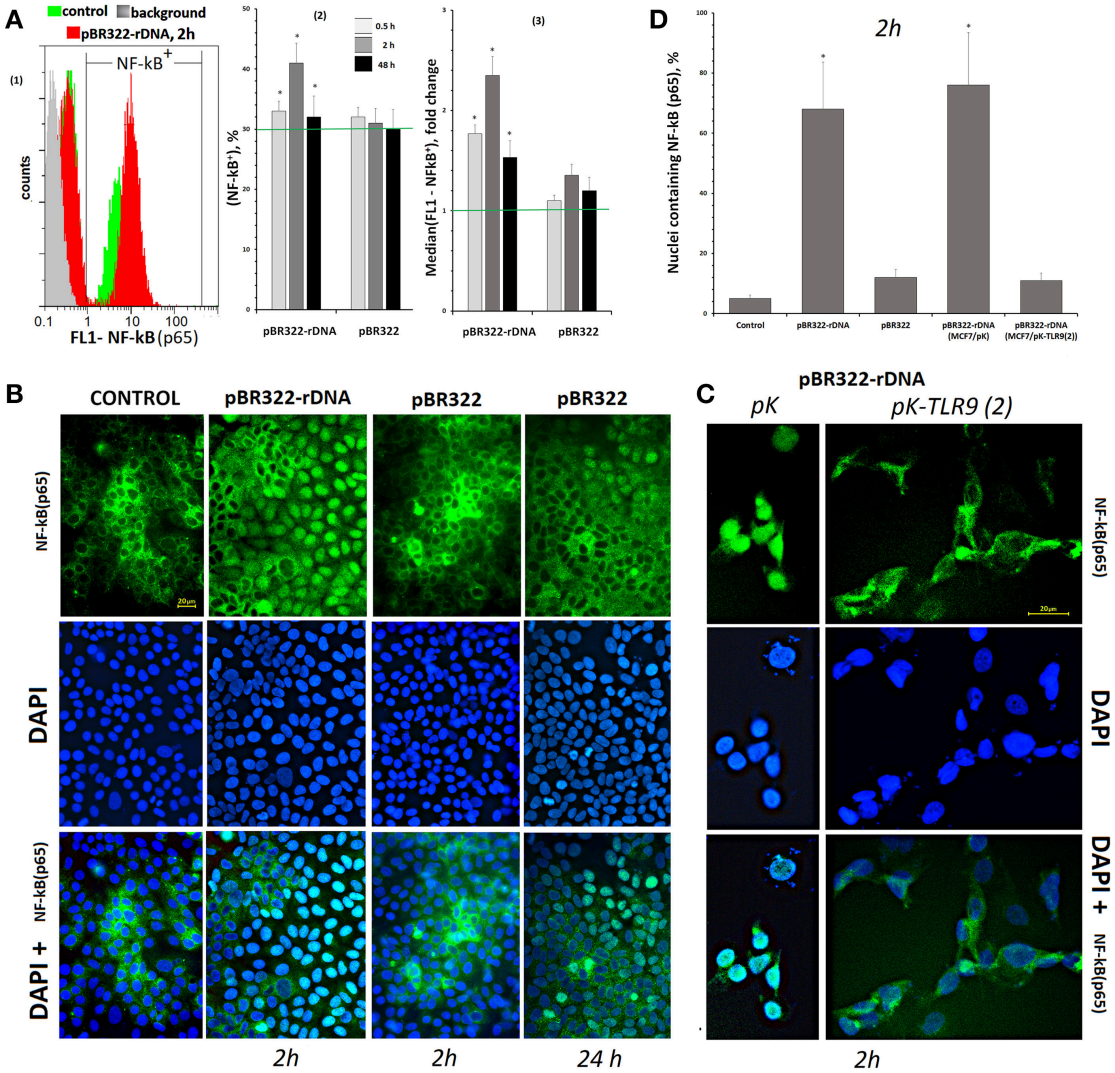
Changes in the activity of NF-κB in MCF7 cells exposed to GC-DNAs. **(A)** FAC. (1) Distribution of fluorescence intensities of the cells stained with NF-kB (p65) antibodies (FITC). (2) The proportion of cells (NF-κB)+ in three studied types of MCF7 cultures. (3) The average of the median signal intensities of FL1 for (NF-κB)+. Each point on the graph is an average of three independent experiments. **(B)** FM of the cells stained with NF-κB (p65) antibodies. The cells were cultured for 24 h, and then GC-DNAs were added for 2 or 24 h. **(C)** Blocking TLR9 inhibits NF-kB activity. We used the cells, which express average amounts of TLR9 protein (24 h of cultivation). Transfection of the plasmids pK and pK-TLR9(2) into the cells was performed with Turbo Fect reagent for 24 h. Two hours after changing the medium to fresh, pBR322-rDNA was added to the cells for 2 h. Under the action of pBR322-rDNA, NF-κB is not activated in the cells containing pK-TLR9(2). **(D)** Graph of the proportion of cells with nuclear staining for NF-κB in studied types of MCF7 cultures. All the FM-experiments **(B,C)** were repeated three times.

Using FCA, we studied changes in the content of NF-kB (p65) protein under the action of GC-DNAs, Figure 4A. In the control cells cultivated for 24 h, two subpopulations can be detected—with high content of the protein (30%) and with low content (70%), Figure 4A(1). Under the action of pBR322—rDNA, the cell count of NF-kB (p65)^+^ fraction [Figure 4A(2)] and mean content of the protein in cells of this fraction [Figure 4A(3)] increased. The vector had virtually no effect on NF-kB (p65)^+^ fraction size, with just a slight augmentation of the protein content in this fraction.

In control cells, which had been cultivated for 24 h, NF-kB was located mostly in the cell cytoplasm, Figures 4B,D. The plasmid that carried an insert of TR-rDNA induced translocation of NF-kB from the cytoplasm to the nucleus in 2 h in 70% of the cells. This fact suggests induction of NF-kB activity in the presence of TR-rDNA fragments. The vector pBR322 did not enhance NF-kB activity: in 2 h, the factor still was located in the cell cytoplasm. In 24 h, translocation of NF-kB to the nucleus was observed in ~15% of the cells.

The translocation of NF-kB into the nucleus under the action of pBR322-rDNA does not occur if the RNA *TLR9* is previously blocked with siRNA (Figure 4C). Two plasmids: pK-TLR9(2) encoding fragments of siRNA for gene *TLR9* and pK plasmid without the insert were used for this experiment. In the treated with pBR322-rDNA population of cells containing pK-TLR9 (2), the number of nuclei containing the factor NF-kB was several times less than in cells transfected with the pK vector (Figure 4D). Thus, it can be assumed that TLR9 stimulation with pBR322-rDNA is the main cause of NF-kB activation.

#### Activation of STAT3 Transcription Factor in the Presence of GC-DNAs

Signal transducer and activator of transcription 3 (STAT3) belongs to the signal transducer and activator of transcription (STAT) family. STAT3 is inactive in non-stimulated cells, but is rapidly activated by various cytokines, such as IL-6 (52). This factor plays a significant role in the resistance of cancer cells to therapy. NF-κB can affect STAT3 activity because NF-κB regulates expression of the STAT3-activating cytokine IL-6.

Plasmid pBR322—rDNA in 2 and 48 h provoked expression of RNA for signal transducer and activator of transcription 3 and 6 (STAT3, STAT6) and genes for metalloproteinases MMP2 and MMP7. Their transcription is under the control of STAT3 (Table 3, lines 24–27). The vector also induced expression of *STAT3, STAT6*, and *MMP7* RNA, but as late as in 48 h.

**Figure 5:**
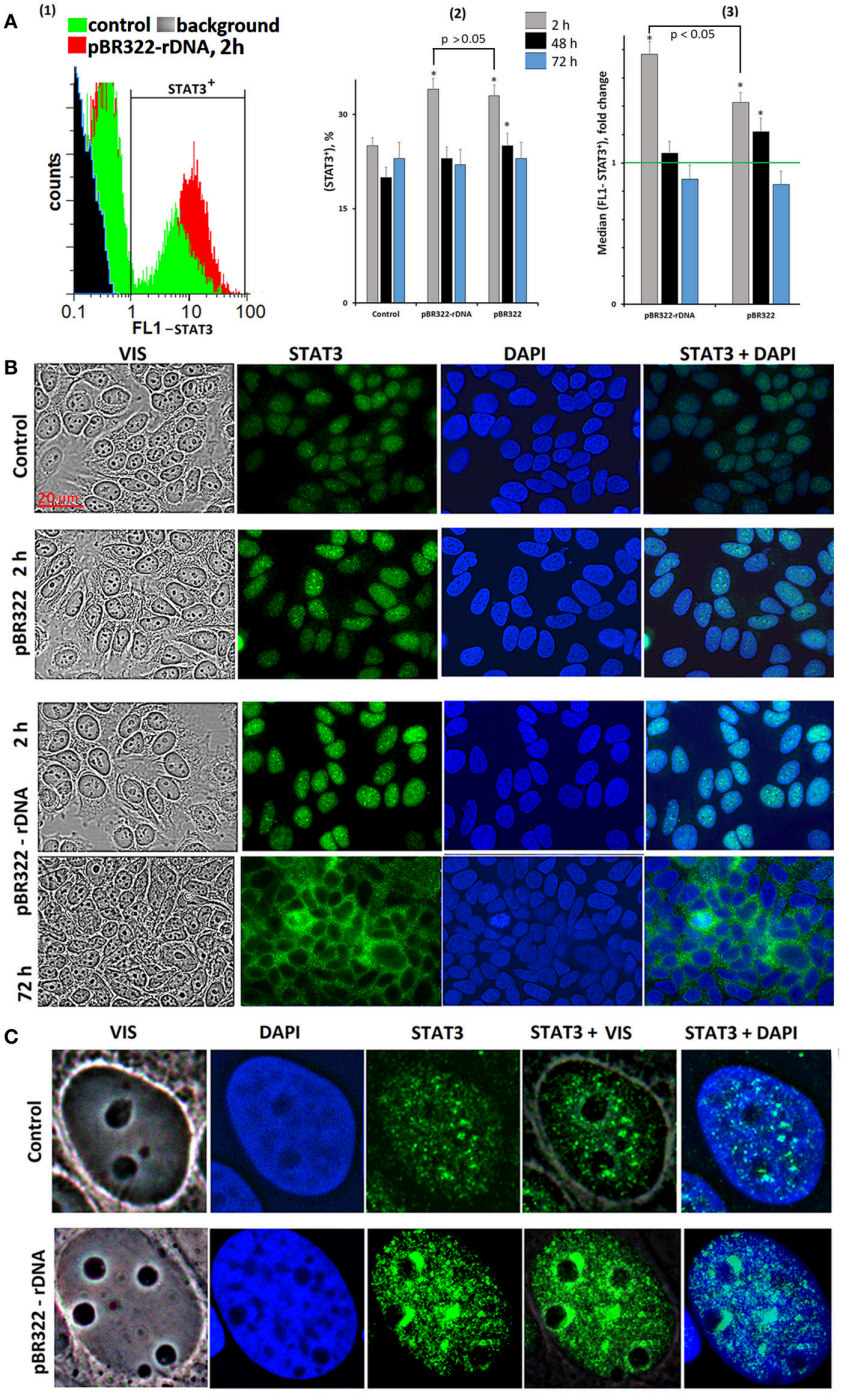
Changes in activity of transcriptional factor STAT3 in MCF7 cells exposed to GC-DNAs. **(A)** FAC. (1) Distribution of fluorescence intensities of the cells stained with STAT3 antibodies (FITC). (2) The proportion of cells (STAT3)+ in three studied types of MCF7 cultures. (3) The average of the median signal intensities of FL1 for (STAT3)+. Each point on the graph is an average of three independent experiments. **(B,C)** FM of the cells stained with STAT3 antibodies. The cells were cultured for 48 h, and then GC-DNAs were added. Experiment was repeated three times.

We assayed STAT3 protein in the cells using FCA and FM, Figure 5. The level of the protein in the STAT3+ fraction increased by 40 and 70% in 2 h of cultivation with, respectively, pBR322 and pBR322—rDNA [Figure 5A(3)]. Inside the cells, STAT3 is located in the nucleus as compact granules, Figures 5B,C. In 2 h of the exposure to the plasmid, the protein content increased. The protein was accumulated in those areas of the nucleus, which encompass the nucleoli. In 72 h, location of the protein essentially changed—STAT3 was detected in the cytoplasm only, suggesting inhibition of the factor.

Thus, *STAT3* expression and STAT3 factor activity after the exposure to GC-DNAs at first increase and then decrease below the baseline.

### TR-rDNA Stimulates DNA Damage Response in MCF7 Cells

DNA damage response is a network of cellular pathways that sense, signal and repair DNA lesions. Stimulation of DDR is important for the survival of cancer cells (53). We have previously shown that oxidized human DNA stimulates the DDR in MCF7 cells (47). The main cause of DNA damage in the cancer cells can be an increased level of ROS. ROS damage DNA by inducing DNA breaks. DNA damage stimulates the DDR, therefore, we analyzed the effect of GC-DNAs on the level of ROS and on the level of DNA breaks in MCF7 cells.

### GC-DNAs Induce ROS Synthesis in MCF7 Cells

**Figure 6:**
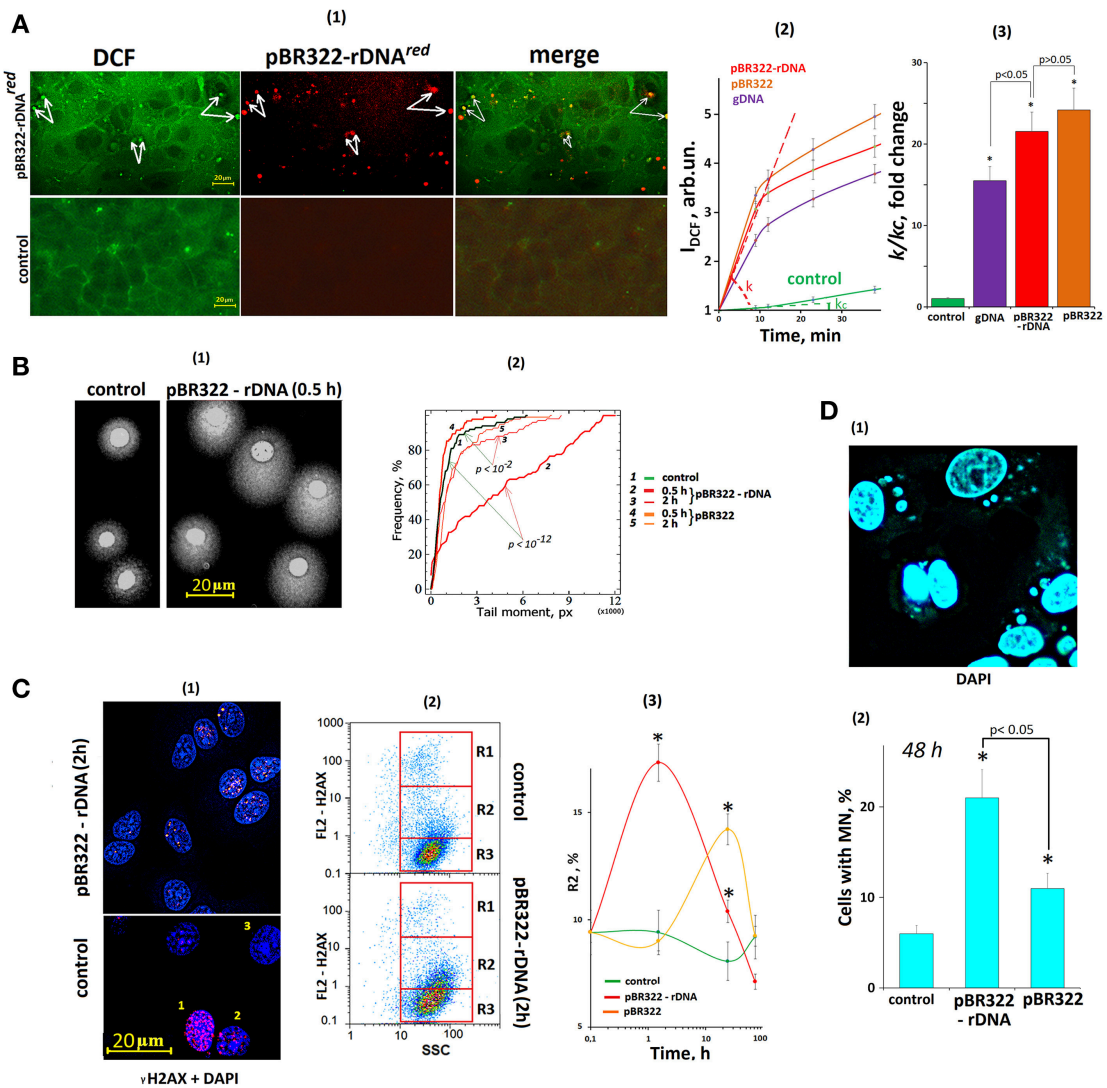
The exposure of MCF7 cells to GC-DNAs leads to an increase in ROS production and DNA damage. **(A)** (1) FM—based evaluation of MCF7 cells treated with 5 μm H_2_DCFH-DA and pBR322–rDNA^*red*^ (50 ng/mL) and incubated for 30 min. Top photo: green granules—DCF and red granules—pBR322–rDNA^*red*^ in the cells. Some of the signals are the same (some examples of overlapping signals are indicated by arrows), indicating DCF synthesis at the site of DNA contact with the cell. The result is reproduced in three independent experiments. (2) FL-plate reader (37°C). The cells were analyzed using total fluorescence assay in the 96-well plate format at λ_ex_ = 488 nm and λ_em_ = 528 nm. Eight repeated measurements were provided for each GC-DNA and 16 for the control. The mean absolute intensities were divided by the average value of the intensity corresponding to *t* = 0, obtaining the values of *I*_0_ = 1. (3) The value of the tangent of the slope (*k*) together with the error of determination was calculated. *k*_c_ = 1—reaction rate constant for DCF formation in the control cells. *p < 0.05 (comparison with the control). **(B)** (1) Comet assay in alkaline conditions. Digital photography of the nuclei with varying degree of DNA damage. (2) Cumulative histograms for the tail moment. Statistics of Kolmogorov–Smirnov are given on the graph. The result is reproduced in three independent experiments. **(C)** dsDNA breaks in the cells exposed to GC-DNAs. Cells were processed for immunofluorescence staining with anti γH2AX antibody (γ-foci). (1) FM. Control and pBR322-rDNA (50 ng/mL, 2 h). Numbers denotes three detected types of nuclei: 1—with multiple dsDNA breaks, 2—with a few dsDNA breaks, 3—with intact DNA. (2) FAC analysis of γ-foci. Fractions of the cells as evident in gating areas R1, R2, R3. (3) Relative proportions of cells within gating area R2. The result is reproduced in three independent experiments. **(D)** Evaluation of micronuclei (MN) in MCF7 treated with GC-DNAs (50 ng/mL, 48 h). (1) Example of DAPI staining of the fixed cells treated with pBR322-rDNA. (2) Proportion of the cells with modified nuclei in three studied types of MCF7 cultures. Viewed 30 photos and determined the average cell content with micronuclei.

The primary MCF7 cell response to a change of cfDNA content in the culture medium is ROS synthesis, Figure 6A. The ROS synthesis was analyzed using reaction of DCF formation (54). After the exposure of the cells to GC-DNAs, the total signal level increased, and isolated brightly stained DCF grains appeared in the cells. Joint allocation of DCF and fluorescently labeled pBR322-rDNA^red^ samples showed that ROS synthesis became intensified in the places of contact between DNA and the cell. Location of some signals from fluorescently labeled DNA (red) and DCF grains (green) coincided (some of these signals are indicated by white arrows, Figure 6A).

The kinetics of DCF formation upon the exposure to GC-DNAs was examined using a fluorescent reader, Figures 6A(2,3). The GC-DNAs induced ROS synthesis to a greater extend, than genomic DNA isolated from the cells. The rate of ROS synthesis began to grow immediately after adding DNA samples to the medium. As quickly as 20 min later, the rate of ROS synthesis considerably reduced. The pBR322—rDNA and pBR322 plasmids stimulated ROS generation with the same effectiveness.

### GC-DNAs Induced DNA Damage in MCF7 Cells

The number of nuclear DNA breaks was evaluated using comet assay (alkaline electrophoresis), Figure 6B. In 30 min after adding pBR322—rDNA to the medium, DNA breaks were detected in the most cells [“comets” were formed, Figure 6B(1)], but 2 h later, the number of breaks considerably reduced. pBR322 also stimulated break formation, but the effect was much weaker Figure 3B(2).

The number of double strand DNA breaks was evaluated via determining the content of H2AX histone in the nuclei, Figure 6C. In the control cells, three types of nuclei occur: with abundant gamma-focuses [type 1, Figure 6C(1)], with single breaks (type 2) and nuclei without DNA breaks (type 3). The numbers of nuclei of each type were determined using FCA, Figure 6C(2). FL2-H2AX—SSC plots can be divided into three areas: with a great number of H2AX (gate R1), with relatively high H2AX content (gate R2), and with low signal level (gate R3). After the cells were exposed to pBR322—rDNA for 2 h, we observed a two-fold increase in the size of fraction 2—cells with single gamma-focuses. The numbers of cells with multiple gamma-focuses did not differ significantly. In case of exposure to pBR322, the increase in the cell fraction R2 was observed as late as after 24 h of the cultivation.

One of the indicators of genome instability due to DNA damage is the formation of micronuclei (MN) (49). GC-DNAs significantly increases the number of cells with micronuclei (Figure 6D).

### GC-DNAs Stimulate DNA Repair Response and Increase Cell Survival

**Figure 7:**
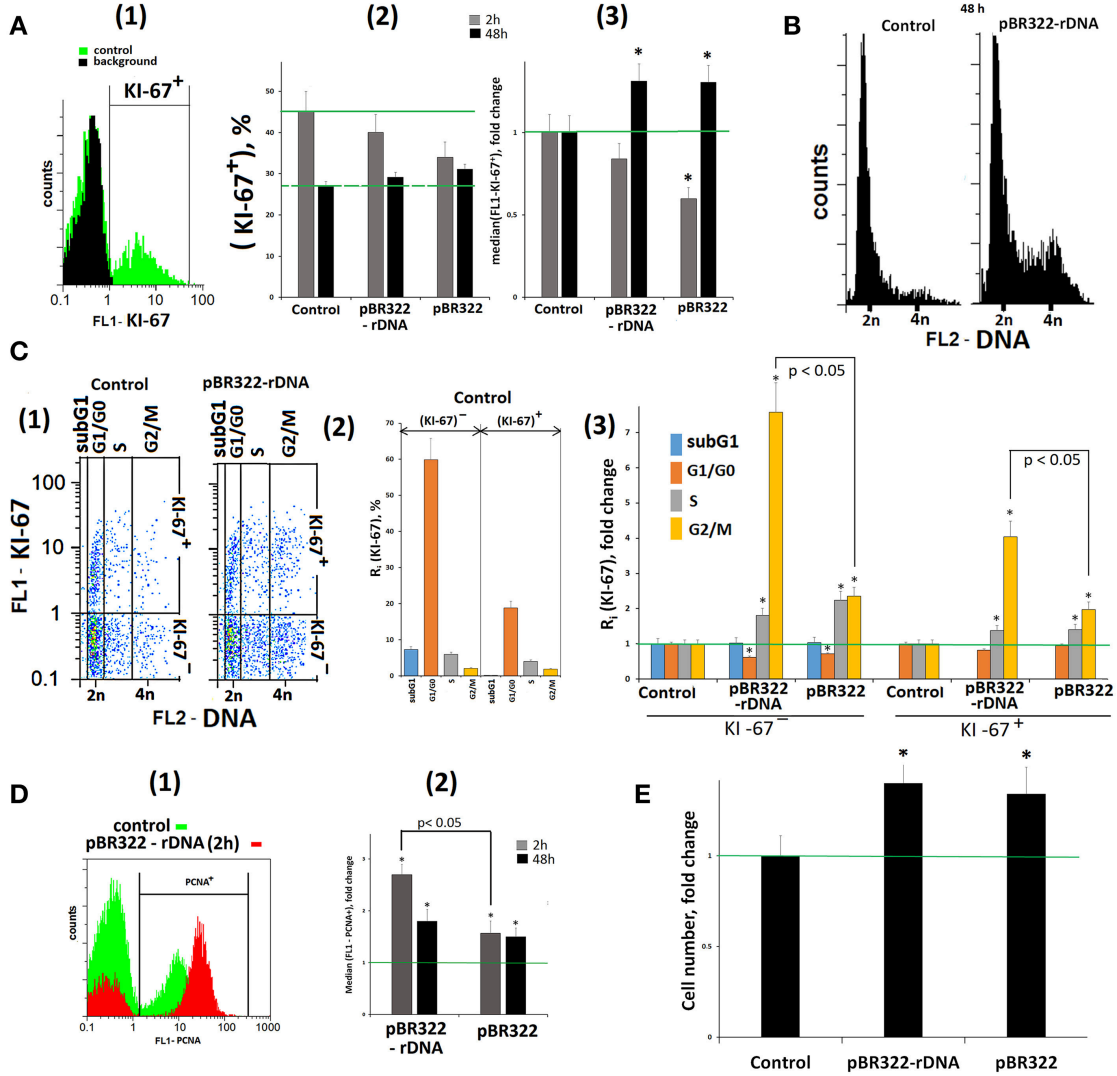
Proliferation and cell cycle of MCF7 cells exposed to GC-DNAs. **(A)** (1) FCA. Fixed cells stained with anti-Ki-67 antibodies (green). Background fluorescence was quantified using FITC-conjugated secondary antibodies (black). (2) Proportion of KI-67+ cells in total cell population. (3) The average (*n* = 3) of the median signal intensities of FL1 (KI-67+). **(B)** FCA. Distribution of fluorescence intensities of the cells stained with PJ (DNA content). **(C)** (1) FCA. Cells plots: FL1-KI-67 vs. FL2-DNA. (2) The content in the KI-67+ and KI-67- control populations cells with the amount of DNA corresponding to the SubG1/G0, G1/G0-, S and G2/M phases of the cell cycle. (3) Effect of the plasmids on the relative proportions of the cell with the amount of DNA corresponding to the phases of the cell cycle. Cultivations conditions: GC-DNAs, 50 ng/mL, 48 h. (2 and 3)—the averaged data for three experiments. **(D)** (1) FCA. Fixed cells stained with anti-PCNA antibodies. (2) The average (*n* = 3) of the median signal intensities of FL1 (PCNA+). **(E)** Total number of cells in studied cell population (50 ng/mL GC-DNAs, 48 h). Before the start of the experiments, cells were cultured for 24 h. *p < 0.05 (comparison with the control).

A transient cell cycle arrest and proliferation blocking is one of the initial stages of a cell's response to DNA damage (53). We determined the relative number of proliferating cells by using antibody to the proliferation marker KI-67 and FCA technique (Figure 7A). In response to GC-DNAs (50 ng/mL, 2 h) the number of (KI-67)^+^ cells [Figure 7A(2)] and the mean signal intensity [Figure 7A(3)] decrease, suggesting the cell proliferation blocking. This line of evidence for a cell cycle arrest was also supported by qRT-PCR analysis of mRNA encoding the inducible cell cycle arrest proteins, including *CDKN2A, CDKN1A, CCND1, TP53*, and an important negative regulator of the p53 tumor suppressor *MDM2* (Table 1, lines 33–36, 8).

In 48 h, the signal intensity of (KI-67)^+^ fraction exceeds the control level, Figures 7A(2,3), and amounts of RNA for genes *CDKN1A, TP53*, and *MDM2* remain elevated. These genes regulate the cell's damage response.

Figure 7B shows the distribution of cells by the amount of DNA in nuclei, stained with propidium iodide. The exposure to GC-DNAs for 48 h leads to the augmentation of the fraction of the cells with an amount of DNA more than 2n (S—and G2/M cycle phases). Figure 7C(1) presents a plot of FL1-KI-67 signal to FL2-DNA signal. Two sub-populations can be isolated: (KI-67)^+^(proliferating cells) and (KI-67)^−^. The histogram Figure 7C(2) displays the content of fractions subG1, G1/G0, S, and G2/M in (KI-67)^+^ and (KI-67)^−^ sub-populations in the control. The relative values of fractions sizes for the cells exposed to GC-DNAs are shown in Figure 7C(3). In both sub-populations, the content of S and G2/M fractions is augmented in the presence of GC-DNAs. In case of pBR322—rDNA, the effect of accumulation of G2/M cells is much stronger, than in case of the vector.

Thus, an increase in the GC-content of extracellular DNA induces an arrest of a part of cells in S– and G2/M–phases of the cell cycle. Cells with an elevated DNA amount accrues in MCF7 population with the time of cultivation.

In response to the DNA break formation in the presence of pBR322–rDNA, the cells demonstrated growing expression of proliferating cell nuclear antigen (PCNA),—a protein that participates in excision DNA repair (data obtained with FCA, Figure 7D).

GC-DNAs stimulate *BRCA1* expression (Table 3, line 1). This gene is directly involved in the repair of double strand breaks. The amount of RNA for *BRCA1* increased twice in 2 h and remained elevated 48 h later.

The improved survival ability in the presence of GC-DNAs is suggested by the increased content of RNAs for some anti-apoptotic genes (*BCL2, BCL2A1, BCL2L1, BIRC2, BIRC3*) (Table 3, lines 3–7). The level of expression is elevated in 2 h after adding pBR322-rDNA to the medium, and 48 h later, the expression of some genes remains heightened (*BCL2, BCL2A1, BCL2L1*, and *BIRC2*). The vector also stimulates the expression of the anti-apoptotic genes, but as late as in 48 h. In parallel with the increase in expression of the anti-apoptotic genes, the levels of expression of pro-apoptotic genes *BAX, BID, BAD, NOXA, BBC3* (lines 37–42) and tumor suppressor genes *HUWE1* and *AMBRA* (lines 9 and 10) decrease in the presence of plasmids pBR322–rDNA and pBR322.

pBR322—rDNA induces an increase in the expression of the regulator of oxygen homeostasis *HIF1-alpha* (Table 3, line 11). In addition, the content of RNA for the genes of Akt/mTOR signaling pathways (*MTOR, AKT1, AKT2, AKT3*, lines 29–32) and the gene for growth factor *VEGFA* (line 28) drastically elevate. In contrast to plasmid pBR322—rDNA, the vector has virtually no effect on the expression of gene *HIF1-alpha*, genes of Akt/mTOR signaling pathways, and *VEGFA*.

The total cell number in the presence of GC-DNAs increased in 48 h of cultivation by 30–40% compared to the control cells (Figure 7E).

## Discussion

Cell free rDNA can be considered as a marker for accumulation of GC-rich fragments of the genome in the pool of cfDNA for some diseases (26–28). These diseases are characterized by an increased level of cell death. Changes in the GC- content of cfDNA leads to the emergence of a new biological activity of this DNA with respect to various body cells. In this study we showed, that (1) fragments of rDNA accumulate in breast cancer patient's cfDNA in appreciable quantity. (2) TR-rDNA fragments in the form of the plasmid pBR322-rDNA stimulate TLR9/MYD88/NF-kB signaling pathway and suppresses expression of AIM2. (3) TR-rDNA fragments induce DDR in MCF7 cancer cells.

### TR-rDNA Fragments Stimulate TLR9/MYD88/NF-kB Signaling Pathway and Suppresses Expression of AIM2

Aberrant expression of TLR9 in tumor cells was shown to be able to promote tumor growth and invasion (39, 40, 43). In the literature, the data of TLR9 expression in MCF7 cells are contradictory. Some authors registered a lack of TLR9 (38), whereas the others detected expression of TLR9 in MCF7 cells (41). According to study (43), TLR9 is expressed both on the surface of MCF7 cells and in the cytoplasm. We have found that the level of TLR9 expression depends on the duration of cell cultivation. The content of TLR9 protein increased by an order after the cell cultivation over 72 h (Supplementary Material). One of the possible explanations is the growth of the content of cfDNA, which can induce expression of *TLR9*. Model GC-DNAs also promoted *TLR9* expression. pBR322—rDNA stimulated *TLR9* expression much higher and faster, than vector pBR322 (Figure 3A).

Activation of TLR9 with the rDNA fragments induces the activity of NF-kB (Figure 4). NF-kB is considered to play key roles in the development and progression of many cancers. Cytokines whose expression is induced in response to NF-kB in immune cells of the tumor microenvironment lead to STAT3 activation in both malignant and immune cells (55, 56). Activation of NF-kB and STAT3 resulting from an exposure to TR-rDNA (Figures 4, 5) can be one of the causes of the cancer cells resistance to the therapy.

Simultaneously with the increasing *TLR9* expression, the expression of intra-cytoplasmic sensors AIM2 is appreciably suppressed (Figures 3A,D). AIM2 is known to play a critical role as a tumor suppressor. Loss of AIM2 expression promotes tumor progression (32–36). AIM2 suppresses human breast cancer cell proliferation *in vitro* and mammary tumor growth in a mouse model (37). The level of *AIM2* expression in case of pBR322—rDNA is declined much more, than in case of pBR322, that corroborates more active stimulation of *TLR9* expression.

In order to examine, to what extent the levels of expression of the two genes for DNA-sensors are associated, we used siRNA (Supplementary Material). The silencing RNA *TLR9* considerably increased both contents of RNA *AIM2* and AIM2 protein. We failed to reveal a pronounced reverse effect: silencing RNA *AIM2* did not result in any substantial alteration in the content of RNA *TLR9*, but the content of TLR9 protein slightly increased.

The biological activity of AIM2 is realized in the cytoplasm via binding to intra-cytoplasmic DNA. This protein is a component of inflammasomes forming in the cytoplasm (57). When the level of *AIM2* expression is high enough (in 24–48 h after the beginning of cultivation), the protein is detected in the cytoplasm. When the level of *AIM2* expression reduces due to the activation of *TLR9* expression, AIM2 protein translocates from the cytoplasm to certain inner nuclear regions. The translocation of AIM2 to the nucleus seems to be one of the ways to diminish activity of this sensor, together with reducing expression of *AIM2* gene.

Thus, DNA-sensors TLR9 and AIM2 perform opposite functions to entail either survival (TLR9) of death (AIM2) of the cancer cells. TR-rDNA fragments can affect NF-kB in two ways: to enhance activity of NF-kB by means of activation of TLR9 and simultaneously to suppress activity of AIM2, a suppressor of NF-kB (56). It seems that blocking the activity of *TLR9* in a course of anticancer therapy can achieve two purposes: (1) reduce the resistance of cancer cells to treatment and (2) promote cell death due to activation of AIM2-associated signaling pathway.

### GC-DNA Fragments Induce DDR in MCF7 Cancer Cells

Cancer cells have high ROS production. At the site of contact between the GC-DNA and the cell membrane, the synthesis of ROS further increases (Figure 6A). Oxidation of cfDNA considerably enhances its ability to penetrate MCF7 cancer cells. The mechanism for penetration is so far obscure. We assume an existence of cell receptors, which recognize the oxidized cfDNA and perform transportation of this DNA into the cells. Therefore, oxidized DNA fragments easily penetrate MCF7 cells and induce ROS synthesis in the mitochondria (47). The explosion of ROS synthesis near the cell nucleus induces DNA damage. In response to DNA damage, the cell activates the DDR.

Activation of the signaling pathways that provide the DNA damage response, as well as activation of TLR9—NF-kB- signaling, contributes to the survival of the cancer cell. In the presence of TR-rDNA fragments (in the form of pBR322-rDNA) in the medium, the cells demonstrate elevated levels of expression of genes for proteins involved in processes that are aimed to cancer cell survival. Such genes include DNA repair genes (*BRCA1, PCNA*), a regulator of oxygen homeostasis (*HIF1-alpha*), anti-apoptotic genes, tumor suppressors, genes of the Akt/mTOR signaling pathway and vascular endothelial growth factor A (*VEGFA)* (Table 3). In addition, TR-rDNA fragments block the expression of pro-apoptotic genes.

It is important to note that both GC-DNAs (pBR322-rDNA and pBR322) induce qualitatively much the same responses in MCF7, aimed to heighten the cancer cell's survivability. The major difference consist in the fact that the cell response to the action of pBR322–rDNA is realized much earlier and with higher efficiency, than the response to the action of pBR322. This can be seen, first of all, when analyzing changes of expression of the genes for cytoplasmic DNA receptors, such as TLR9, AIM2 (Figure 3) and STING (Table 1). We explain this fact primarily by a high oxidation rate of the insert in pBR322-rDNA compared to the vector in the conditions of a short-time increase in ROS production at the moment of interaction with the cell surface.

### The Specificity of the Cell Response to the Action of rDNA Fragments

The region of TR-rDNA as a part of pBR322–rDNA also harbors a sequence, which determines specificity of the cell response to TR-rDNA fragments only. When studying the expression of *TLR9* induced by the presence of GC-DNAs in the culture medium (Figure 3), we revealed an interesting fact. TLR9 protein, the content of which becomes considerably increased after the exposure to pBR322–rDNA (or pEFGP-rDNA), can be found in the area of nucleoli, where the ribosomal genes are located. As vector pBR322 (or pEFGP) do not induce accumulation of TLR9 in the nucleolus, one can suppose that they are rDNA fragments, which provide for the TLR9 transportation to and fixation in the nucleolus. This intriguing fact requires further research. TLR9 might not be a unique protein transported by rDNA fragments to the nucleolus. It was shown, that rDNA locus could titrate genome-wide levels of various factors (58, 59). Besides, the fact of finding out TLR9 in the nucleolus suggests, that rDNA fragments reach the nucleolus. The presence of additional rDNA fragments in the nucleolus can result in binding the transcription factors and affect the ribosome biogenesis.

The plasmid pBR322–rDNA greatly enhances expression of genes of the Akt/mTOR signaling pathway (Table 3, lines 29–32), whereas the vector pBR322 does not have this ability. mTOR activity is known to be associated with the ribosome biogenesis. In cancer cells, activation of Akt/mTOR signaling pathway resulting in heightening the metabolic rate and cancer cell proliferation is observed (60). mTOR protein can bind to rDNA. Perhaps, rDNA facilitates nuclear translocation of the factors that enhance the expression of genes of the Akt/mTOR signaling pathway.

**Figure 8:**
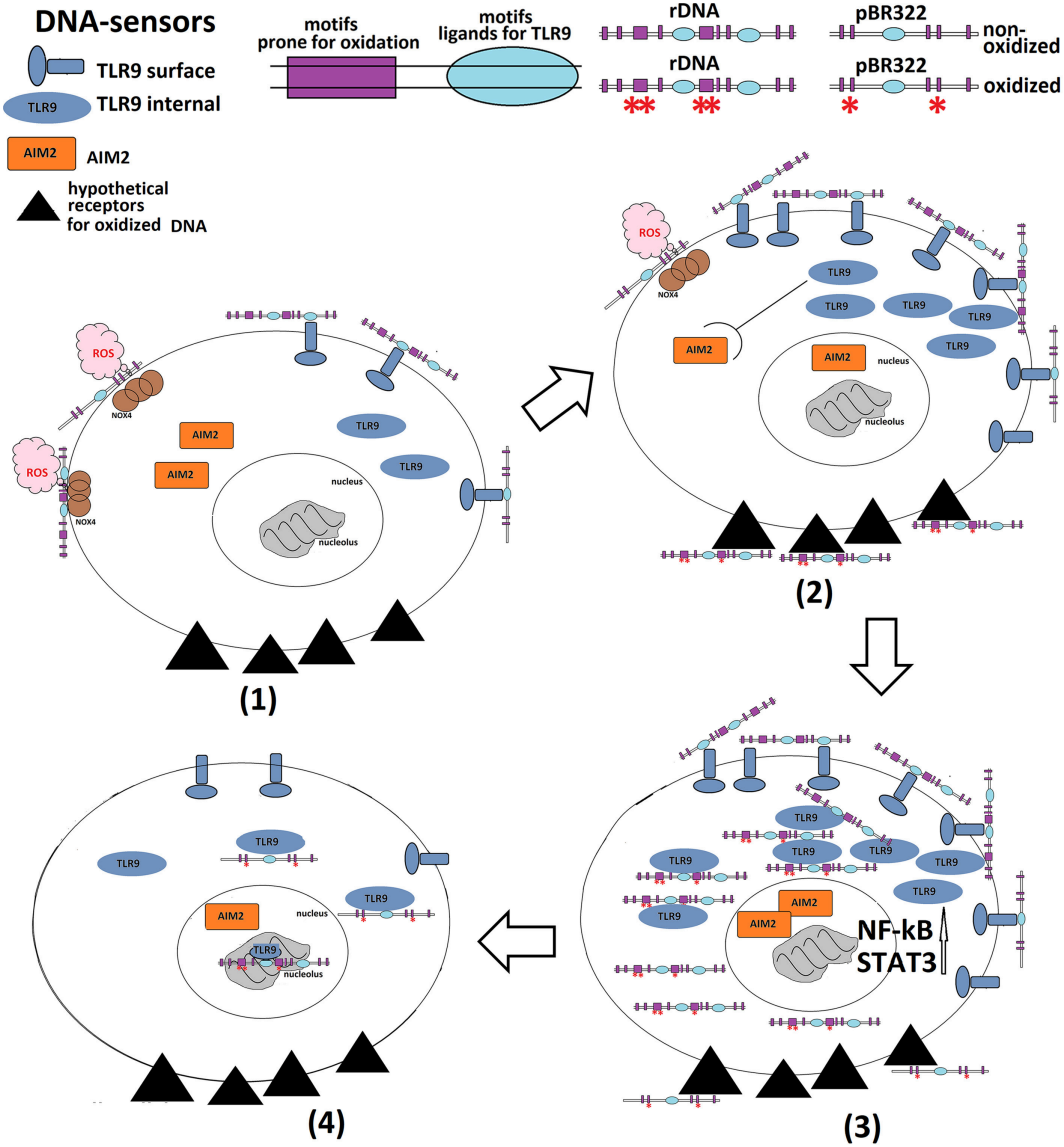
A summary of the events developing in MCF7 cells exposed to GC-DNAs. *Early response (first few minutes – first hours of exposure)*. (1) GC-DNAs interact with the cell surface and stimulate ROS synthesis at the site of contact. GC-DNAs also interact with the surface TLR9. (2) GC-DNAs are oxidized with the ROS. The oxidation level depends on the GC-DNA sequence. Vector pBR322 is oxidized slowly. The rDNA fragments rapidly oxidize and interact with hypothetical sensors of oxidized DNA. TLR9 expression increases significantly and AIM2 expression is blocked. Proliferation is temporarily blocked. (3) Oxidized rDNA fragments penetrate the cell and interact with the internal TLR9s. TLR9-MyD88-NF-κB and STAT3—signaling is stimulated. DNA-sensors AIM2 are completely blocked: protein expression in the cytoplasm is reduced, the protein moves into the nucleus. Oxidized DNA fragments stimulate oxidative stress in mitochondria (47), ROS level in the nucleus increases temporarily. DDR is developing. Expression of the repair genes, antiapoptotic genes, gene of the mTOR- signaling is increased. *Late response (24 h or more)*. (4) Fragments of oxidized rDNA penetrate the nucleolus in the form of complexes with TLR9. The pBR322 fragments oxidize and penetrate into the nucleus, causing a response similar to rDNA. Apoptosis is blocked. The total number of cells is increased compared to the control. The cancer cells with an unstable genome survive. Many cells in the state of G2/M arrest.

Thus, we have shown that extracellular TR-rDNA is a biologically active molecule, which can affect the functional activity of MCF7 cancer cells (Figure 8). The biological activity of extracellular TR-rDNA in human body is suggested by the occurrence of specific antibodies we found in human blood serum (29). Antibodies against TR-rDNA exist in a free form or bound to cfDNA. The constant for the interaction of antibodies with TR-rDNA is by an order of magnitude higher than the constants for conventional standard DNA antibody complexes with dsDNA in SLE (systemic lupus erythematosus). Moreover, serum of healthy donors contains virtually no conventional antibodies to dsDNA. The immune system of a healthy control seems to produce specific antibodies to TR-rDNA for the purpose of destroying and/or eliminating this active DAMPs molecule from the blood stream. It can be speculated that an intake of the antibodies to TR-rDNA for the removal of extracellular TR-rDNA from the cancer patient's body might contribute to more effective tumor destruction during the therapy.

## Conclusion

In cfDNA derived from breast cancer patients, the content of TR-rDNA is increased several times compared to controls. TR-rDNA fragments can act as DAMPs for MCF7 cancer cells. TR-rDNA induces TLR9/MYD88/NF-kB and DDR- signaling pathways and suppresses expression of AIM2. The exposure of cells to extracellular TR-rDNA leads to elevated expression of genes responsible for cancer cell survivability. As a result, in MCF7 population cell are accruing with instable and changed genome. Cell-free TR-rDNA can promote transportation of some proteins to the nucleolus. It is expedient to regard cell free TR-rDNA as a possible object of the cancer therapy.

## Ethics Statement

The investigation was carried out in accordance with the latest version of the Declaration of Helsinki. The Regional Ethics Committee of RCMG approved it (approval #5). All participants signed an informed written consent to participate after the nature of the procedures had been explained to them.

## Author Contributions

SVK, NV, VV, and SIK designed the study. EM, ES, EE, EK, MK, and NO performed the experiments. NV and LP performed the statistical analysis. EM provided cell cultures. SVK, VV, and NV wrote the initial draft. LP translated the manuscript to English. All the authors participated in critical revision and approved the final manuscript before submission.

### Conflict of Interest Statement

The authors declare that the research was conducted in the absence of any commercial or financial relationships that could be construed as a potential conflict of interest.

## Supplementary Material

Supplementary Figure 1 Genes *AIM2* and *TLR9* respond to a change in the time of cultivation in different ways: *TLR9* expression considerably increases, while *AIM2* expression drastically decreases. **(A)** FCA of AIM2 and TLR9 expression in MCF7. Cells were stained with AIM2 or TLR9 antibody (FITC). Plots: FL1 (TLR9 or AIM2) vs. SSC. The MCF7 population includes the cells with a high content of TLR9 (30%) and the cell with a low content. In both subgroups, TLR9 expression grows up during cultivation. The percentage of the cells with a high TLR9 content increases up to 60% in 72 h. Expression of AIM2 protein also changes during cultivation. The cells can be divided in two fractions—with the high AIM2 content (40%), and with the lower AIM2 content. The amount of AIM2 protein in cell culture grows up in 48 h, but after that, it decreases back as fast as in 24 h. In 96 h, the content of AIM2 decreases by an order of magnitude compared to the baseline value in the start of cultivation. **(B)** The dependence of the median signal intensity FL1 (TLR9 or AIM2) (1), the RNA (TLR9 or AIM2) content (2) and the ratio FL1/RNA (3) on the time. With time of cell cultivation, the fraction of RNA *TLR9* considerably grows up. The (TLR9 protein) /(RNA *TLR9*) ratio achieves the maximum as fast as in 24 h and holds constant further. RNA *AIM2* significantly decreases in 72 h of cultivation. The (AIM2 protein)/(RNA *AIM2*) ratio increases during the first 2 days of cultivation, and decreases after that. ^*^ *p* < 0.05 - against control cells, non-parametric U-test.

Supplementary Figure 2 The dependence of the cfDNA concentration on the duration of the cultivation for the control cells.

Supplementary Figure 3 Inhibiting TLR9 and AIM2 expression with the siRNAs. Four plasmids [pK-TLR9(1), pK-TLR9(2), pK-AIM(1), and pK-AIM(2)] encoding fragments of siRNA for genes TLR9 and AIM2 were used (Table 1). The control is a pK plasmid without the insert. We used the cells, which express maximum amounts of AIM2 protein and average amounts of TLR9 protein (24–48 h of cultivation). Transfection of the plasmids into the cells was performed with Turbo Fect reagent. **(A)** RT-qPCR. Estimation of the amount of the RNA *TLR9* and *AIM2*. **(B)** FCA. Estimation of the amount of the proteins TLR9 and AIM2. Either TLR9 expression inhibitor [(pK-TLR9(1) and pK-TLR9(2)] lowered several times the content of RNA *TLR9* as compared to the plasmid—vector pK. The content of TLR9 protein also decreases, but merely by 30% (when pK-TLR9(2) was used). Plasmids [(pK-TLR9(1) and pK-TLR9(2)], while suppressed expression of RNA *TLR9*, substantially stimulated expression of RNA *AIM2* (by a factor of 4-6) and, to a smaller degree, expression of AIM2 protein (by 40–50 %). Inhibitors of *AIM2* expression [pK-AIM2(1) and pK-AIM2(2)] reduced the levels of both RNA *AIM2* (1.5–2 times) and AIM2 protein (by 30–40%). At the same time, the content of RNA *TLR9* changed insignificantly, and the TLR9 protein content slightly increased by 20–40%. Thus, inhibition of *TLR9* expression considerably elevates *AIM2* expression, especially at the level of RNA amount. Inhibition of *AIM2* expression affects *TLR9* expression to a smaller degree. * *p* < 0.05 - against control cells, non-parametric U-test.
