# Supplementary figures and images for: Ribosomal DNA as DAMPs Signal for MCF7 Cancer Cells
Source: Front Oncol. 2019 May 30;9:445. doi: 10.3389/fonc.2019.00445 (PMC6552851; doi:10.3389/fonc.2019.00445)

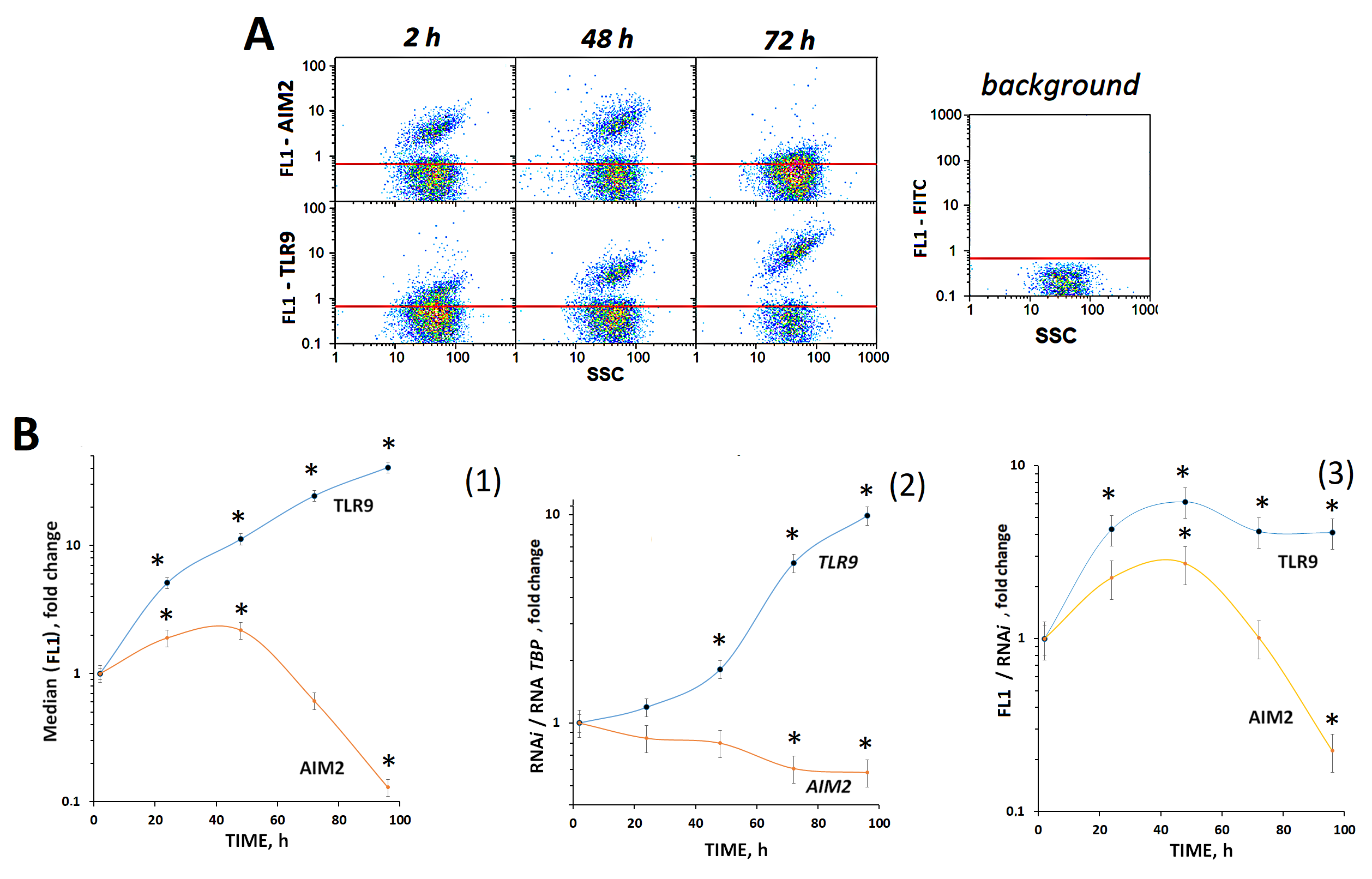

Supplement: Supplementary Figure 1 — Genes AIM2 and TLR9 respond to a change in the time of cultivation in different ways: TLR9 expression considerably increases, while AIM2 expression drastically decreases. (A) FCA of AIM2 and TLR9 expression in MCF7. Cells were stained with AIM2 or TLR9 antibody (FITC). Plots: FL1 (TLR9 or AIM2) vs. SSC. The MCF7 population includes the cells with a high content of TLR9 (30%) and the cell with a low content. In both subgroups, TLR9 expression grows up during cultivation. The percentage of the cells with a high TLR9 content increases up to 60% in 72 h. Expression of AIM2 protein also changes during cultivation. The cells can be divided in two fractions—with the high AIM2 content (40%), and with the lower AIM2 content. The amount of AIM2 protein in cell culture grows up in 48 h, but after that, it decreases back as fast as in 24 h. In 96 h, the content of AIM2 decreases by an order of magnitude compared to the baseline value in the start of cultivation. (B) The dependence of the median signal intensity FL1 (TLR9 or AIM2) (1), the RNA (TLR9 or AIM2) content (2) and the ratio FL1/RNA (3) on the time. With time of cell cultivation, the fraction of RNA TLR9 considerably grows up. The (TLR9 protein) /(RNA TLR9) ratio achieves the maximum as fast as in 24 h and holds constant further. RNA AIM2 significantly decreases in 72 h of cultivation. The (AIM2 protein)/(RNA AIM2) ratio increases during the first 2 days of cultivation, and decreases after that. * p < 0.05 - against control cells, non-parametric U-test. [file Image_1.TIF]

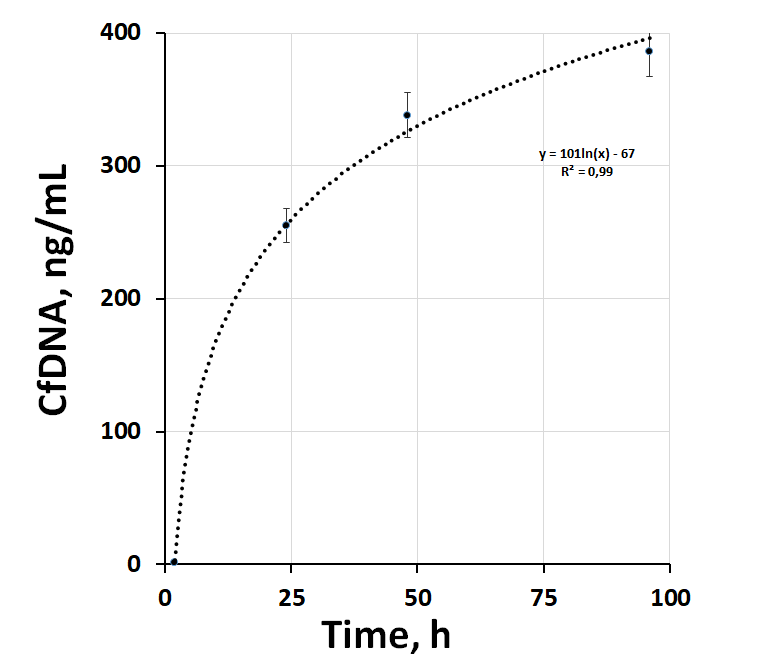

Supplement: Supplementary Figure 2 — The dependence of the cfDNA concentration on the duration of the cultivation for the control cells. [file Image_2.TIF]

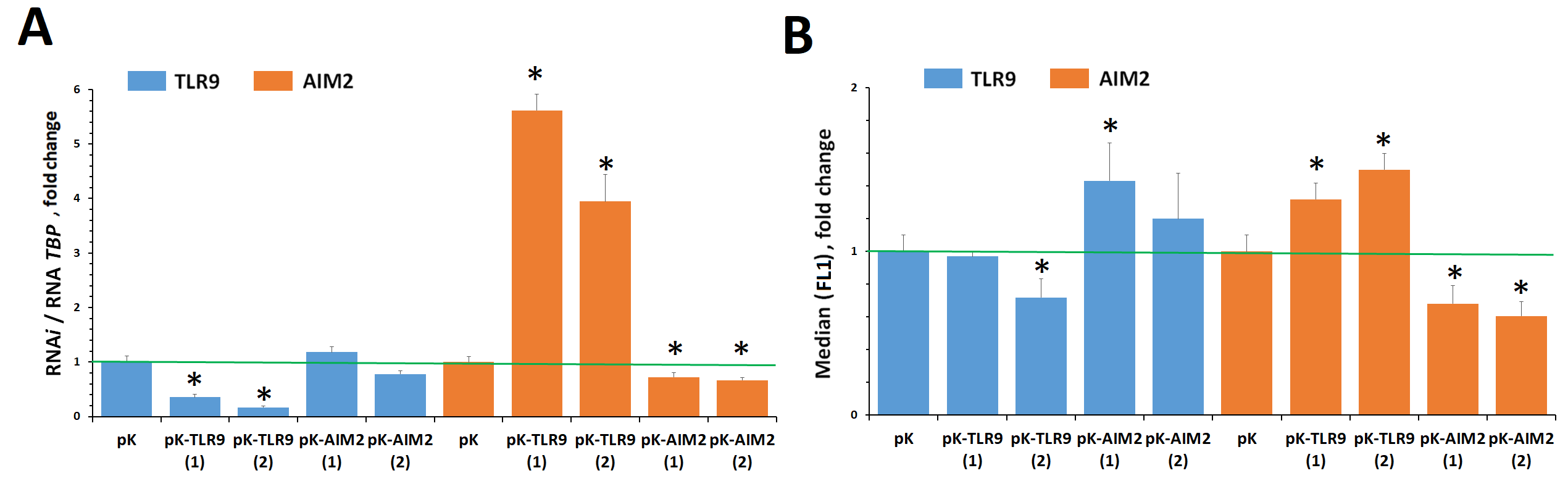

Supplement: Supplementary Figure 3 — Inhibiting TLR9 and AIM2 expression with the siRNAs. Four plasmids [pK-TLR9(1), pK-TLR9(2), pK-AIM(1), and pK-AIM(2)] encoding fragments of siRNA for genes TLR9 and AIM2 were used (Table 1). The control is a pK plasmid without the insert. We used the cells, which express maximum amounts of AIM2 protein and average amounts of TLR9 protein (24–48 h of cultivation). Transfection of the plasmids into the cells was performed with Turbo Fect reagent. (A) RT-qPCR. Estimation of the amount of the RNA TLR9 and AIM2. (B) FCA. Estimation of the amount of the proteins TLR9 and AIM2. Either TLR9 expression inhibitor [(pK-TLR9(1) and pK-TLR9(2)] lowered several times the content of RNA TLR9 as compared to the plasmid—vector pK. The content of TLR9 protein also decreases, but merely by 30% (when pK-TLR9(2) was used). Plasmids [(pK-TLR9(1) and pK-TLR9(2)], while suppressed expression of RNA TLR9, substantially stimulated expression of RNA AIM2 (by a factor of 4-6) and, to a smaller degree, expression of AIM2 protein (by 40–50 %). Inhibitors of AIM2 expression [pK-AIM2(1) and pK-AIM2(2)] reduced the levels of both RNA AIM2 (1.5–2 times) and AIM2 protein (by 30–40%). At the same time, the content of RNA TLR9 changed insignificantly, and the TLR9 protein content slightly increased by 20–40%. Thus, inhibition of TLR9 expression considerably elevates AIM2 expression, especially at the level of RNA amount. Inhibition of AIM2 expression affects TLR9 expression to a smaller degree. * p < 0.05 - against control cells, non-parametric U-test. [file Image_3.TIF]
